# Photoresponsive prodrug‐dye nanoassembly for in‐situ monitorable cancer therapy

**DOI:** 10.1002/btm2.10311

**Published:** 2022-03-12

**Authors:** Kaiqi Long, Yifan Wang, Wen Lv, Yang Yang, Shuting Xu, Changyou Zhan, Weiping Wang

**Affiliations:** ^1^ State Key Laboratory of Pharmaceutical Biotechnology The University of Hong Kong Hong Kong China; ^2^ Department of Pharmacology and Pharmacy, Li Ka Shing Faculty of Medicine The University of Hong Kong Hong Kong China; ^3^ Laboratory of Molecular Engineering and Nanomedicine, Dr. Li Dak‐Sum Research Centre The University of Hong Kong Hong Kong China; ^4^ Department of Pharmacology, School of Basic Medical Sciences & State Key Laboratory of Molecular Engineering of Polymers Fudan University Shanghai China; ^5^ School of Pharmacy, Fudan University & Key Laboratory of Smart Drug Delivery, Ministry of Education Fudan University Shanghai China

**Keywords:** drug delivery, in‐situ monitoring, IR783 dye, nanomedicine, photocleavable prodrug, photopharmacology, self‐assembly

## Abstract

Photocleavable prodrugs enable controllable drug delivery to target sites modulated by light irradiation. However, the in vivo utility is usually hindered by their insolubility and inefficient delivery. In this study, we report a simple strategy of co‐assembling boron‐dipyrromethene‐chlorambucil prodrug and near‐infrared dye IR783 to fabricate photoresponsive nanoassemblies, which achieved both high prodrug loading capacity (~99%) and efficient light‐triggered prodrug activation. The incorporated IR783 dye not only stabilized the nanoparticles and contributed tumor targeting as usual, but also exhibited degradation after light irradiation and in‐situ monitoring of nanoparticle dissociation by fluorescent imaging. Systemic administration of the nanoparticles and localized light irradiation at tumor sites enabled monitorable and efficient drug release in vivo. Our results demonstrate that such prodrug‐dye co‐assembled nanomedicine is a promising formulation for photoresponsive drug delivery, which would advance the translation of photoresponsive nanomedicines.

## 1 INTRODUCTION

Prodrug strategy has presented potentials in improving the efficacy of chemotherapeutic drugs by conjugating them with functional moieties.^1^, ^2^ So far, prodrugs that respond to various stimuli, including pH,^3^, ^4^, ^5^, ^6^ enzyme,^7^, ^8^ ultrasound,^9^ heat^10^, ^11^ and light,^12^, ^13^, ^14^, ^15^ have been developed to reduce the drug toxicity toward normal tissues while retaining their activity at diseased lesions. Light is one of the most convenient and effective triggers that can be spatiotemporally controlled with both high accuracy and low expense.^16^ Recently, photoresponsive prodrugs have been developed with tailor‐made structures containing photocleavable moieties such as coumarin and boron‐dipyrromethene (BODIPY) for photoactivable chemotherapy.^17^, ^18^, ^19^ For instance, BODIPY‐chlorambucil (BC) prodrug, of which the BODIPY group can be efficiently cleaved upon light irradiation, was developed and achieved effective antitumor effect with the utilization of laser light.^20^ However, due to the hydrophobicity of most photocleavable groups, water solubility of the reported photoresponsive prodrugs is generally poor, which hindered their utility in vivo.^21^, ^22^

To solve the problem, prodrug‐based nanoparticles, which exhibits good water dispersibility, long‐term stability and long circulation time, have been developed.^23^, ^24^, ^25^ Several strategies are promoted to fabricate prodrug‐based nanoparticles, such as enclosing prodrugs into nanoparticles or adjusting prodrugs with a proper hydrophilic‐to‐hydrophobic ratio for self‐assembly.^26^, ^27^ For example, hydrophobic photoresponsive prodrugs can be adjusted to amphiphilic molecules by modifying their photocleavable group to a hydrophilic one, which is conducive to molecular self‐assembly and the formation of prodrug‐based nanoparticles.^18^, ^28^ Moreover, co‐assembly is also a potent method to fabricate nanomedicines.^29^, ^30^ Different molecules can self‐assemble into particles through various intermolecular driving forces, such as hydrophobic interaction, electrostatic interaction, etc. The resulted nanoassemblies present serval advantages like facile preparation, safe metabolism, minimal carrier toxicity and thus, convenient co‐delivery of different therapeutic agents.

**FIGURE 1:**
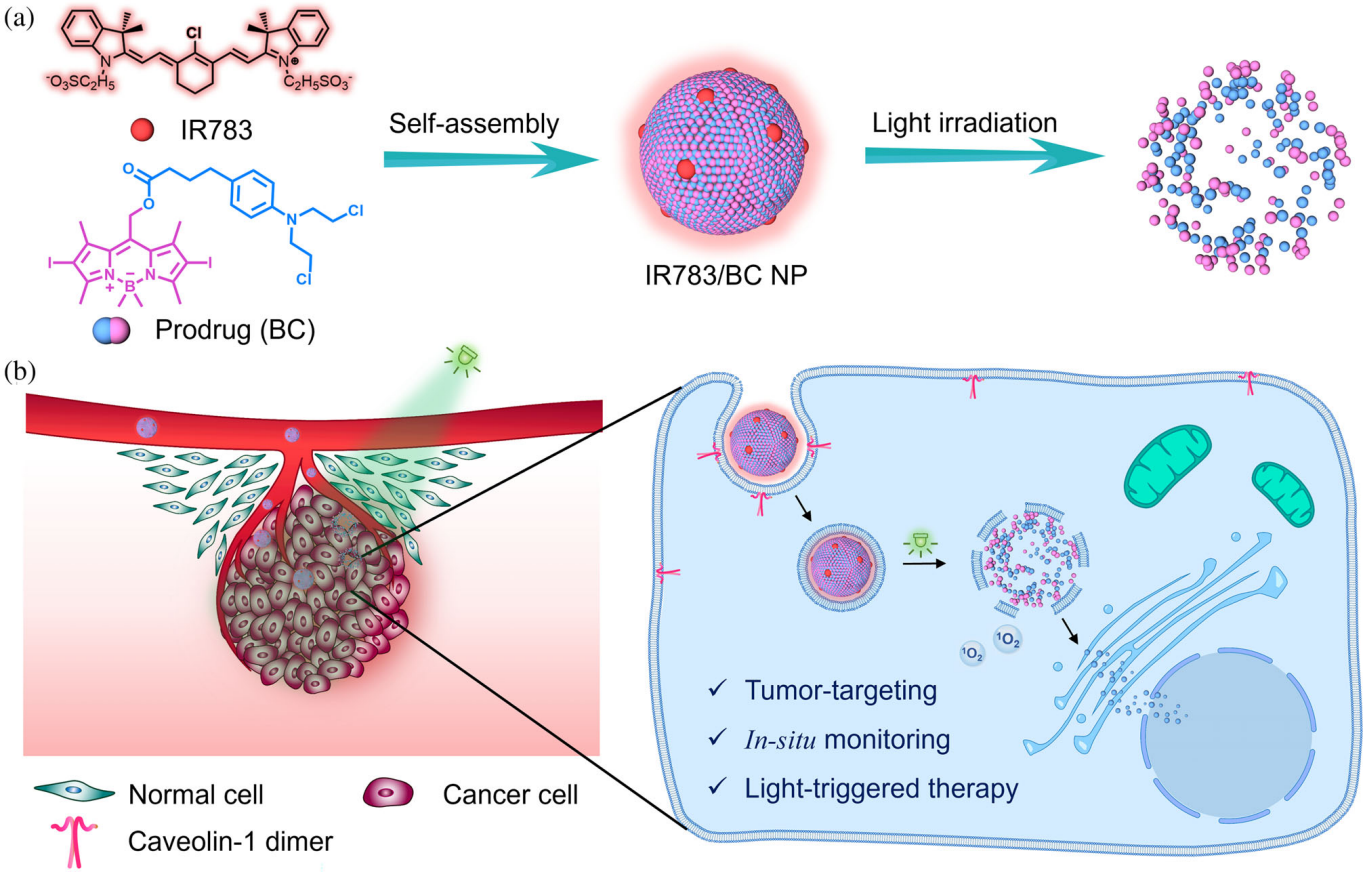
Schematic illustration of photoresponsive IR783/BC NP and its therapeutic effect upon light irradiation. (a) Self‐assembly of IR783/BC NP and its dissociation upon light irradiation. (b) Cellular uptake of IR783/BC NP by a HCT116 cell and light‐triggered drug release for cancer therapy

Here, we design and fabricate a photoresponsive prodrug‐dye nanoassembly for cancer therapy. IR783, a commercially available dye, has been reported to serve as the stabilizer while forming nanoassemblies with hydrophobic drugs.^31^, ^32^, ^33^ In this study, IR783 co‐assembles with photocleavable BC prodrug into prodrug‐dye nanoparticles (IR783/BC NPs; Figure 1a). IR783/BC NPs present great potential for light‐controllable drug delivery based on the photocleavage reaction of BC upon light irradiation, followed by the disassembly of the nanoparticles and the release of free chlorambucil (Cb) drug. Interestingly, IR783 in the nanosystem displays multiple functions by serving as (1) a stabilizer that can be degraded after light irradiation; (2) a targeting moiety that enhances the caveolin‐1 (CAV‐1) mediated transcytosis in tumors; (3) a fluorescent imaging agent for in‐situ monitoring of the disassembly of nanoparticles. Thus, IR783/BC NPs can achieve light‐controllable, tumor‐targeting and in‐situ monitorable cancer therapy (Figure 1b). The efficacy of our system was verified both in vitro and in vivo and the drug release process can be monitored by a in vivo imaging system. The strategy of integrating IR783 and photocleavable prodrug into a multifunctional nanomedicine provides a novel insight for diagnosis and remotely controllable therapy of cancers.

## 2 RESULT AND DISSCUSSION

### 2.1 Preparation and characterization of prodrug‐dye nanoparticles

**FIGURE 2:**
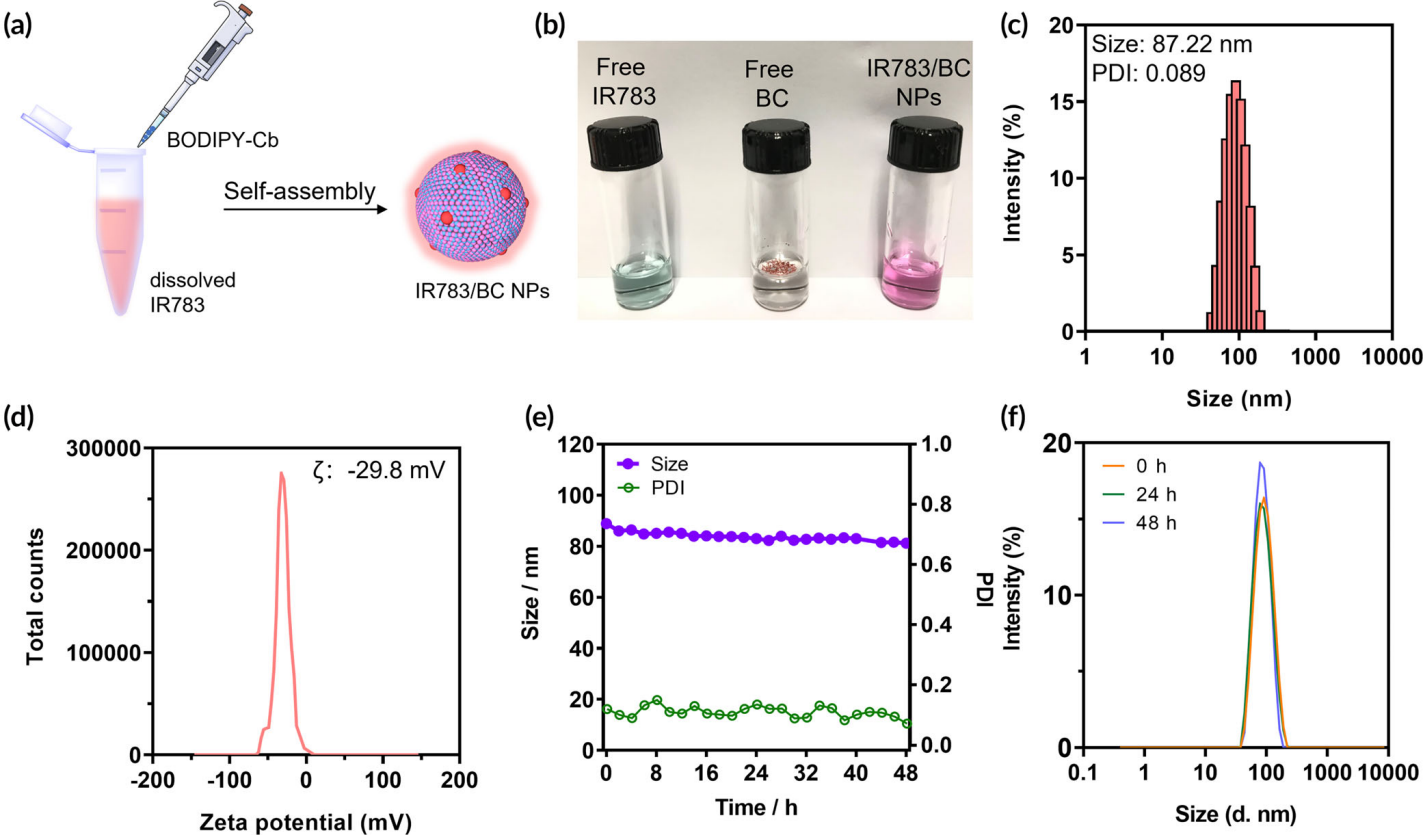
Preparation and characterization of IR783/BC NPs. (a) Nanoparticle preparation by the flash nanoprecipitation method. (b) Representative images of free IR783, free BC prodrug, and IR783/BC NPs in PBS. (c) Representative size and (d) zeta potential distribution of IR783/BC NPs. (e) Stability test of IR783/BC NPs at 37°C in PBS for 48 h. (f) Size distributions of IR783/BC NPs at 0, 24, and 48 h

The synthesis of photoresponsive BC prodrug followed the published method.^34^ As illustrated in Figure 2a, the BC prodrug could co‐assemble with IR783 when injecting the stock solution of BC dropwise in the aqueous solution of IR783 by flash nanoprecipitation method. The excessive IR783 in the solution was removed by centrifugation, and IR783/BC NPs were finally obtained as red‐purple dispersion after resuspension in phosphate buffer saline (PBS) (Figure 2b). We first optimized the feeding ratio of IR783 to BC prodrug by adjusting the concentrations of IR783 solution. When the concentration of IR783 solution was 400 μg/mL, the obtained nanoparticles showed both smallest diameter and lowest polydispersity index (PDI; Figure S1), indicating an optimized mass ratio. Dynamic light scattering (DLS) detected IR783/BC NPs as nanoassemblies with a hydrodynamic diameter of 87.22 nm and a PDI of 0.089 (Figure 2c). The IR783/BC NPs were negatively charged at −29.70 mV, which attributed to the negatively charged sulfonate groups of IR783 (Figure 2d). Besides, IR783 is important for assembling nanoparticles. BC prodrug alone, which is highly hydrophobic and water‐insoluble, could not form well‐dispersed nanoparticles in the aqueous solution while using the same preparation method as IR783/BC NPs (Figure S2). We then quantified the content of IR783 and BC prodrug in the nanoparticles by high‐performance liquid chromatography (HPLC) and calculated the loading capacity and encapsulation efficiency of the two components. As shown in Table S1, we found that both the loading capacity and encapsulation efficiency of BC prodrug were remarkably high, reaching 98.85% and 84.37%, respectively, which demonstrated that BC prodrug was the main component that assembled into IR783/BC NPs in the presence of small amount (~1%) of IR783.

Subsequently, we investigated the stability of IR783/BC NPs in PBS, DMEM medium, fetal bovine serum (FBS)‐containing medium and water, separately, at 37 °C for 2 days in the dark environment. Under such conditions, IR783/BC NPs maintained their diameters and PDI, and the size distributions at different time points are almost the same, indicating that the prodrug‐dye nanoparticles exhibited good stability in various solutions (Figure 2e,f; Figures S3 and S4). The size of nanoparticles in the serum‐containing medium increased from about 120 to 145 nm, because the formation of protein corona stayed in a dynamic status and influenced the size of nanoparticles.^35^

### 2.2 IR783/BC NPs dissociated and released free drug upon light irradiation

**FIGURE 3:**
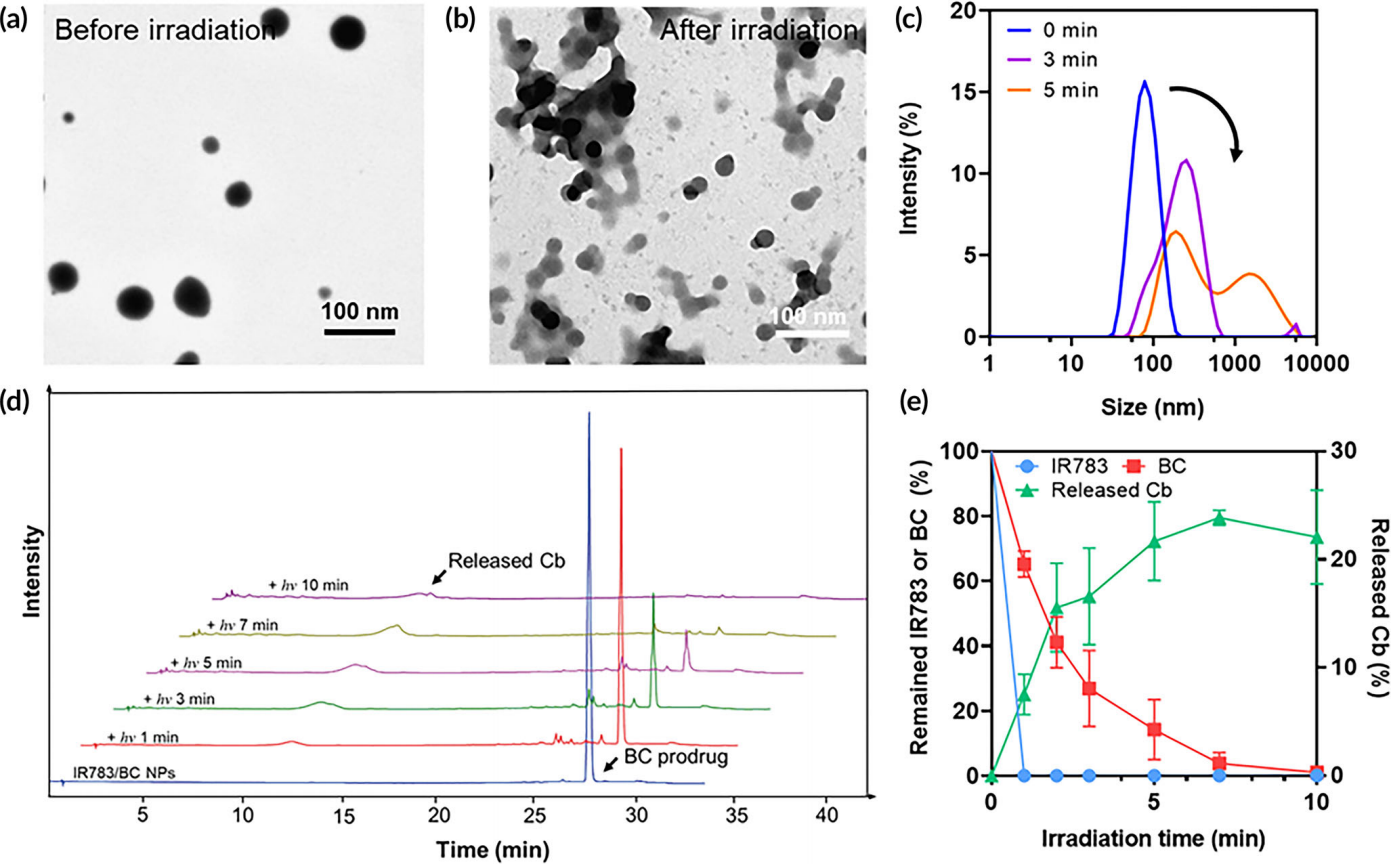
Light‐triggered photocleavage, nanoparticle dissociation and drug release. (a) TEM image of IR783/BC NPs without light irradiation. (b) TEM image of IR783/BC NPs after light irradiation for 5 min. (c) Size distributions of IR783/BC NPs under light irradiation for 0, 3, and 5 min. (d) HPLC analysis of IR783/BC NPs upon light irradiation. (e) Quantitative analysis of IR783/BC degradation and Cb release (*n* = 3). Light irradiation: 530 nm, 50 mW/cm^2^

For practical applications in drug delivery, the photoresponsive nanoparticles need to remain intact in the formulated solution and blood circulation, and intelligently respond to light irradiation for controllable drug release.^36^ Therefore, the photoresponsive property of IR783/BC NPs was investigated by monitoring the size, morphology, and composition changes via DLS, transmission electron microscopy (TEM) and HPLC testing, respectively. It was observed that the spherical IR783/BC NPs dissociated after light irradiation and subsequently formed both large aggregations and small fragments (Figure 3a,b). The hydrodynamic diameter of IR783/BC NPs increased and formed a new peak at about 1000 nm after 530 nm light irradiation at 50 mW/cm^2^, indicating the disassembly of the nanoparticles and the formation of large aggregates (Figure 3c; Figure S5). Moreover, the release process of anticancer drug Cb from BC in IR783/BC NPs upon light irradiation (530 nm, 50 mW/cm^2^) was investigated by HPLC (Figure 3d,e). We noticed that the elution peak at 28.9 min corresponding to the BC prodrug decreased over time. A new broad peak at around 12.0 min corresponding to free Cb gradually increased, indicating the photocleavage of BC prodrug and the release of free drug. Within 10‐min light irradiation, nearly 100% of BC prodrug was degraded. The release percentage of Cb was raised to 23.85% in the first 7 min and then decreased to 22.05% at 10 min. The result can be explained by the fact that some byproducts were generated during the photocleavage reaction and the released Cb was not stable in aqueous solutions (Figure S6).^20^, ^34^, ^37^ These results demonstrated that IR783/BC NPs exhibited both good stability in the dark and excellent photocleavable ability upon light irradiation, confirming their potency for photoresponsive drug delivery.

### 2.3 Light‐triggered dissociation of IR783/BC NPs was monitorable by fluorescence measurement

**FIGURE 4:**
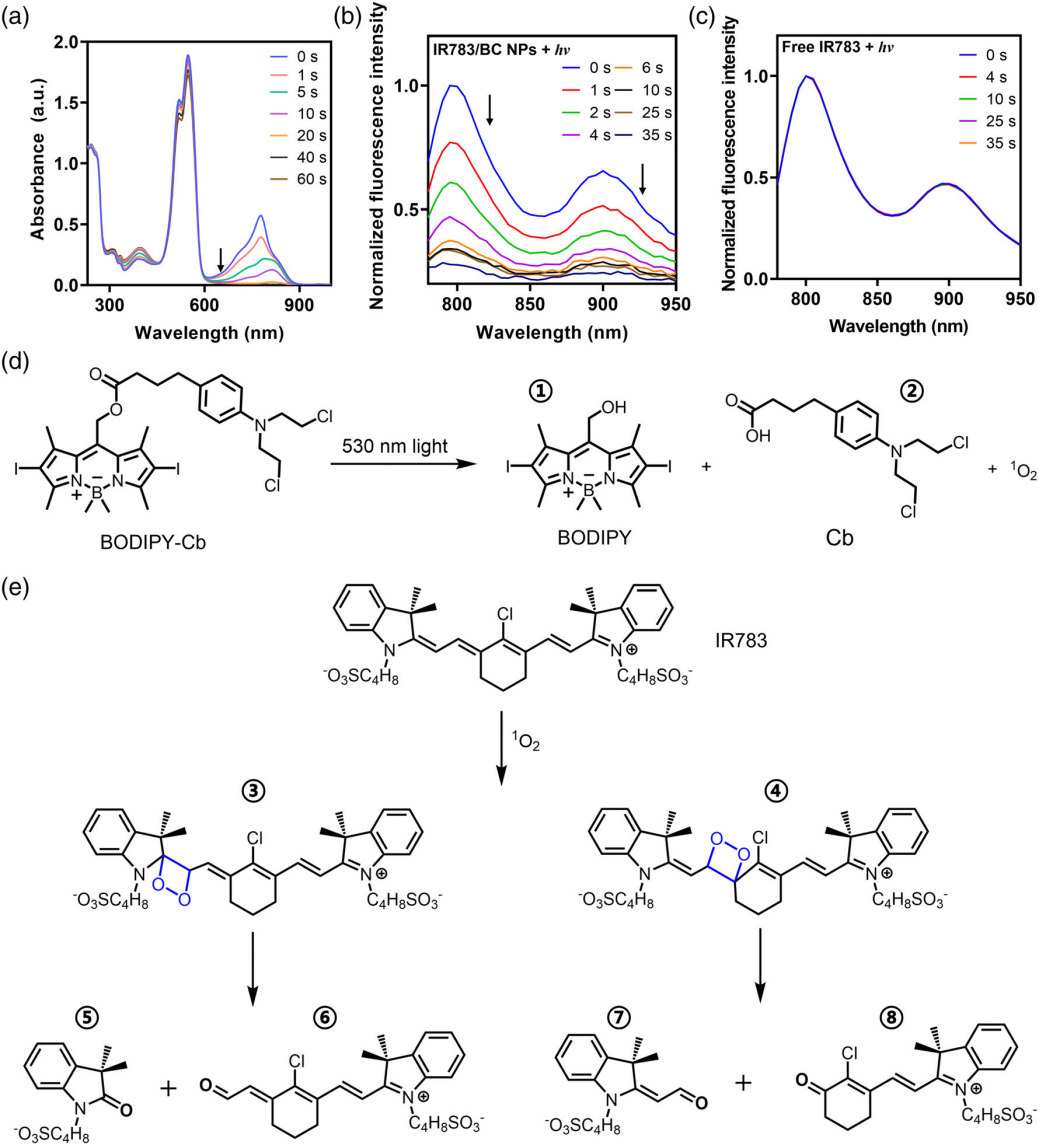
Spectrum study and photochemistry mechanisms. (a) UV‐Vis absorption spectra of IR783/BC NPs upon 530 nm light irradiation. Fluorescence spectra of (b) IR783/BC NPs and (c) free IR783 upon 530 nm light irradiation (Ex. 783 nm). (d) Photocleavage process of BC prodrug. (e) ROS‐triggered decomposition of IR783. The LC‐MS characterization of intermediates and products ①–⑧ is showed in Figure S8. Light irradiation: 530 nm, 50 mW/cm^2^

The photoresponsive property of IR783/BC NPs was further investigated by UV‐Vis‐NIR spectrometry. As shown in Figure 4a, IR783/BC NPs without light irradiation displayed intense absorption peaks at around 530 and 780 nm, which are corresponding to the absorption peaks of BC prodrug and IR783, respectively.^34^, ^38^ Upon light irradiation with a 530 nm LED lamp (50 mW/cm^2^), the absorption peak at 780 nm disappeared within 20 s, indicating the degradation of IR783 in the nanoparticles upon the light irradiation. HPLC result also showed that IR783 in the nanoparticles degraded completely after 1‐min light irradiation (Figure 3e; Figure S7). In Figure 4a, the peak at 540 nm only displayed slight decrease, since the mixture of BODIPY and Cb has similar absorption with BC, which leads to no significant change of the UV‐Vis spectra. Additionally, IR783/BC NPs showed broad NIR fluorescent emission in the region of 780–950 nm that belongs to the emission profile of IR783 dye.^39^ The NIR emission displayed an “ON‐to‐OFF” pattern while applying 530 nm light irradiation on IR783/BC NPs, which also indicated the degradation of IR783 (Figure 4b). Notably, we did not see similar phenomena while free IR783 was irradiated (Figure 4c). Thus, the photoactivation of BC that generates reactive oxygen species (ROS) should play a vital role in the light‐triggered IR783 degradation. It was reported that the cyanine dyes, such as the derivatives of IR783, underwent photooxidation that resulted in the degradation of the cyanine structure.^40^ As the literatures and the above results indicated, IR783 was synchronously degraded during the photocleavage process of BC under green‐light irradiation, the schematic illustrations of the mechanisms were shown in Figure 4d,e. Liquid chromatography‐mass spectrometry (LC‐MS) was used to analyze the residues of IR783/BC NPs after light irradiation and all the intermediates and products were found (Figure S8). The resulted “ON‐to‐OFF” fluorescence switching pattern may enable the in‐situ monitoring of the photoresponsive process of IR783/BC NPs, including accumulation in tumors and light‐triggered dissociation of nanoparticles.

### 2.4 IR783/BC NPs were taken up by HCT116 cells and efficiently induced apoptosis upon light irradiation

**FIGURE 5:**
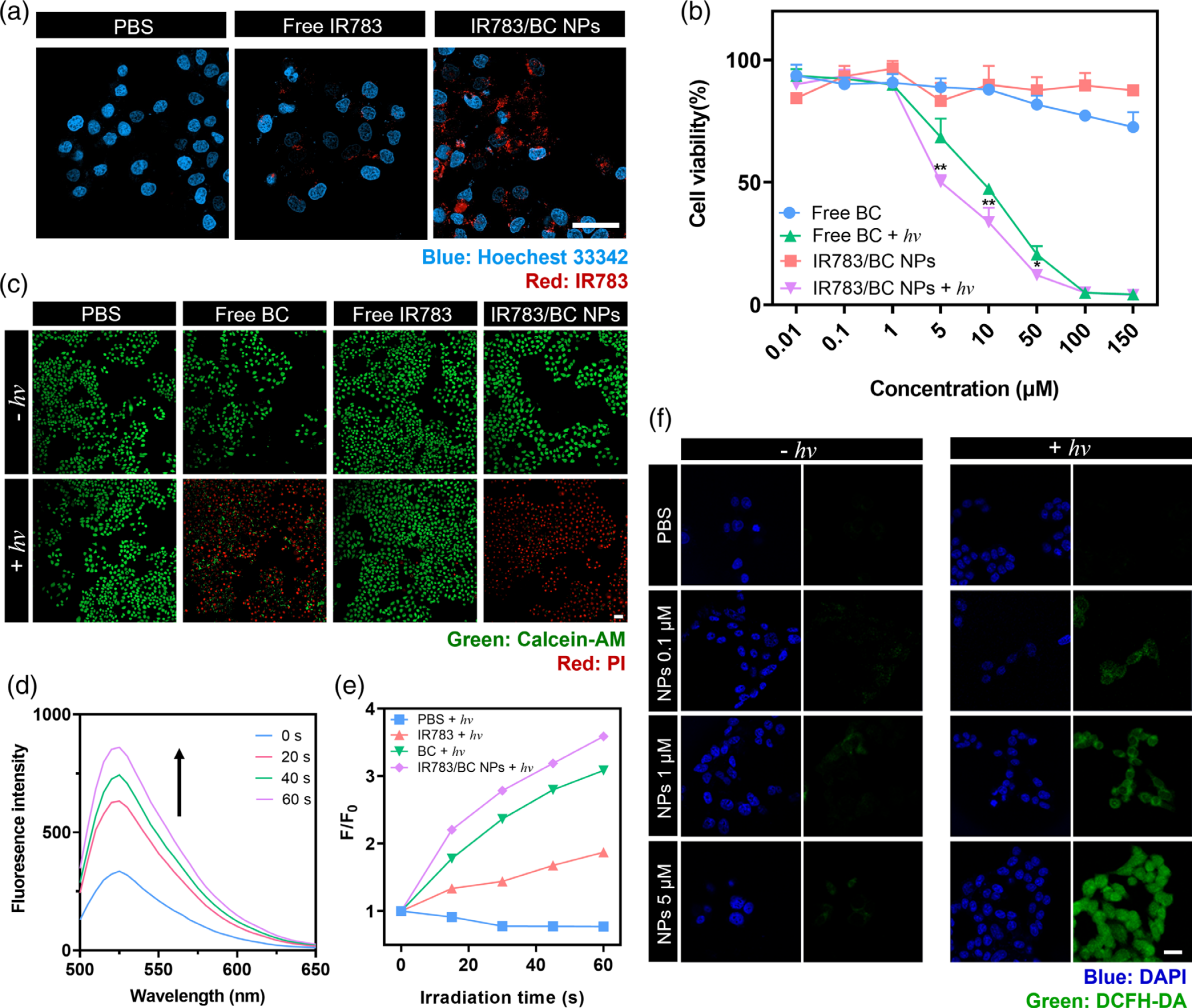
In vitro cellular uptake, cytotoxicity and ^1^O_2_ generation. (a) CLSM images of HCT116 cells incubated with free IR783 and IR783/BC NPs for 6 h. Scale bar: 20 μm. Blue channel: ex. 405 nm; Red channel: ex. 639 nm, with a 650 nm optical filter. (b) In vitro cytotoxicity test of free BC prodrug and IR783/BC NPs with/without light irradiation by MTT assay. *p < 0.05, **p < 0.01. (c) Calcein‐AM/PI staining of HCT116 cells after treatment with free BC, free IR783, IR783/BC NPs with/without light irradiation. Scale bar: 100 μm. (d) Fluorescent change of IR783/BC NPs solution containing SOSG as ^1^O_2_ sensor. (e) The ^1^O_2_ production of IR783, free BC, and IR783/BC NPs in the presence of light. (f) CLSM images of intracellular ^1^O_2_ in HCT116 cells incubated with IR783/BC NPs at different concentrations with/without light irradiation. DCFH‐DA was used as the indicator. Scale bar: 10 μm. Light irradiation: 530 nm, 50 mW/cm^2^, 5 min (except d and e)

The intracellular uptake of IR783/BC NPs in human colon tumor HCT116 cells was evaluated by confocal laser scanning microscopy (CLSM). HCT116 cell line was reported to display high expression of CVA‐1, which can be the target for IR783/BC NPs.^31^, ^41^, ^42^, ^43^, ^44^ As shown in Figure 5a, after 6‐h incubation, the nanoparticles exhibited strong intracellular red fluorescence that represented IR783. Such result was also verified by flow cytometry (Figure S9). Moreover, IR783 was reported as a tumor‐targeting cyanine dye.^45^ Based on previous literatures, the nanoparticles coated by indocyanines with sulfonate groups presented caveolae‐targeting effect, which was confirmed by the result that both caveolin inhibition and CAV‐1 knockout can attenuate the uptake of IR783‐incorparated nanoparticles.^31^ Similarly, the caveolae‐targeting effect of IR783 is also attributed to the efficient cellular uptake of IR783/BC NPs in our study. It should be noted that different cellular uptake in non‐cancerous cells, including human umbilical vein endothelial cells (HUVECs) and human embryonic kidney 293 (HEK 293) cells, as well as some cancer cell lines (Hela cells, HCT116 cells, MCF7 cells, and A549 cells) were observed. The results showed that the endothelial cells (HUVECs) and colon cancer cells (HCT116) exhibited higher cellular uptake of IR783/BC NPs as compared to the other types of cells (Figure S10a,b). Furthermore, the CAV‐1 level of these cell lines were tested by western blotting. Both HUVECs and HCT116 cells showed high CAV‐1 expression (Figure S10c), which is consistent with the previous studies.^41^, ^43^ In all, these results demonstrate that CAV‐1 expression is related to the cellular uptake of IR783/BC NPs.

Furthermore, some internalization pathway inhibitors, including chlorpromazine (clathrin‐mediated endocytosis inhibitor), genistein (caveolae‐mediated endocytosis inhibitor) and m‐β‐cyclodextrin (mβ‐CD) (lipid rafts‐mediated endocytosis inhibitor), and low‐temperature (4°C) incubation were used to analyze the internalization mechanism of IR783/BC NPs. Both genistein and 4°C incubation significantly attenuated the cellular uptake of the nanoparticles, indicating the internalization process dominated by ATP‐dependent and caveolae‐mediated endocytosis (Figure S11). Besides, after the internalization, the red signal of IR783 was co‐localized with the Lysotracker green signal. The fluorescent signal can stay inside lysosomes for at least 24 h (Figure S12). After light irradiation, the IR783 signal decreased, indicating that light irradiation can trigger the degradation of IR783, which was consistent with the result shown in Figure 4b. In vitro cytotoxicity studies including the evaluation of biocompatibility and anti‐proliferation efficiency of our system were investigated. Regarding the increasing concerns about utilizing the synthesized agents in biological systems, the cytotoxicity of IR783, BC prodrug and IR783/BC NPs in the dark was evaluated for the biocompatibility evaluation. Negligible cytotoxicity was observed when the HUVECs were incubated with IR783, BC or IR783/BC NPs separately in the dark for 24 h with a high concentration up to 100 μM, indicating the good biocompatibility of the IR783/BC NPs and their components in the dark (Figure S13a).

Subsequently, the anti‐proliferation effect was evaluated with HCT116 cells by MTT assay (Figure 5b). Upon 530 nm light irradiation, the cell viability decreased gradually to nearly 0% by increasing the concentration of IR783/BC NPs, with an IC_50_ value of 6.62 μM on BC prodrug basis. The nanoparticles exhibited higher therapeutic effect than free BC prodrug under light irradiation (IC_50_ value: 9.24 μM). The cytotoxicity of the free anticancer drug Cb was demonstrated to be relatively low at the experimental conditions (Figure S13b). It should be noted that Cb was observed to rapidly hydrolyze in aqueous solutions, resulting in the loss of its cytotoxicity.^20^, ^46^ Thus, the prodrug strategy effectively enhanced the therapeutic efficacy of Cb. These cytotoxicity results verified that IR783/BC NPs exhibited enhanced therapeutic effect against HCT116 cancer cells upon light irradiation.

To further investigate the therapeutic effect of the nanoparticles, live‐dead staining analysis was conducted by Calcein AM/PI co‐staining. As shown in Figure 5c, the dead cells presenting red fluorescence were observed both in the irradiated BC‐treated group and IR783/BC NPs‐treated group while other groups without light irradiation did not cause significant cell death, which coincided well with the result of the cytotoxicity study. Additionally, apoptosis study of HCT116 cells treated with free Cb or IR783/BC NPs was conducted by Annexin‐V FITC/PI assay to investigate the anticancer activity (Figure S14). Cell apoptosis rate was significantly elevated after treating with IR783/BC NPs and light irradiation. At the concentration of 20 μM, the nanoparticles plus light irradiation induced 77.13% of cell apoptosis, which was mainly dominated by the late apoptosis (70.45%). In comparison, free Cb at the same concentration only caused about 5% apoptosis no matter whether the light irradiation was applied or not, since the free Cb cannot enter the cells as efficient as the nanoparticles. In all, these results confirmed the effective cytotoxicity and apoptosis‐inducing effect of IR783/BC NPs with light irradiation.

### 2.5 IR783/BC NPs generated ROS upon light irradiation

Interestingly, compared with free Cb, the BC prodrug irradiated with 530 nm light showed higher cytotoxicity. One of the reasons should be that the iodide‐containing BODIPY group of BC prodrug exhibits high inter‐system crossing efficiency and enhances singlet oxygen (^1^O_2_) production ability upon light irradiation.^47^, ^48^ For confirmation, ^1^O_2_ generation of IR783/BC NPs was determined by the Singlet Oxygen Sensor Green® (SOSG) Assay. SOSG is a commonly used indicator of ^1^O_2_ that can unequivocally indicate the ^1^O_2_ generation via tunable photoinduced electron transfer mechanism.^49^ As shown in Figure 5d, in the solution containing SOSG and IR783/BC NPs with light irradiation (530 nm, 50 mW/cm^2^, 0–1 min), the fluorescence of SOSG significantly increased in the range of 500–600 nm, indicating the ^1^O_2_ generation from IR783/BC NPs with light irradiation. In comparison, we also observed that the generation of ^1^O_2_ from free BC prodrug was slightly lower than that of IR783/BC NPs at the equivalent concentration of BC (Figure 5e). It was attributed to the fact that the nanoparticles dispersed better than the free BC prodrug in the aqueous solution. The result also showed that the free IR783 dye also generated ^1^O_2_ but at a low efficiency, which is due to the fact that IR783 has low absorbance at 530 nm (Figure S15). Therefore, the observed photodynamic effect of BC prodrug in IR783/BC NPs should also contribute to the cytotoxicity of the nanoparticles upon light irradiation, which could enable efficient chemo‐photodynamic combination therapy by the IR783/BC NPs upon light irradiation.

The intracellular ^1^O_2_ generation by IR783/BC NPs was investigated in HCT116 cells by DCFH‐DA (2,7‐dichlorodihydrofluorescein diacetate). DCFH‐DA is non‐fluorescent in nature whereas forming dichloro‐fluorescein with strong fluorescence in the existence of ^1^O_2_. As shown in Figure 5f, HCT116 cells co‐incubated with both IR783/BC NPs and DCFH‐DA exhibited little green fluorescence, indicating negligible ^1^O_2_ generation in the dark. After 10‐min irradiation at 50 mW/cm^2^, strong intracellular green fluorescence was observed by confocal imaging, which demonstrated the intracellular ^1^O_2_ generation by IR783/BC NPs upon light irradiation. The quantitative measurement of the intracellular ROS generation was conducted with flow cytometry (Figure S16). HCT116 cells were incubated with IR783/BC NPs (5 μM) for 2 h and DCFH‐DA (10 μM) for 30 min before light irradiation. It was observed that the fluorescence increased after light irradiation, indicating the intracellular ROS generation.

Moreover, IR783 is a photothermal agent for photothermal therapy.^50^, ^51^ The photothermal effect of IR783 in nanoparticles was evaluated and compared with water and free IR783. IR783/BC NPs solution was heated up slightly at the first 30 s and cooled down thereafter, while the temperature of free IR783 solution consistently increased by about 6°C after 10‐min light irradiation (Figure S17). Such phenomenon indicated IR783 in IR783/BC NPs was degraded upon light irradiation. Moreover, it was observed that after light irradiation the free IR783 solution remained its green color, while IR783/BC NPs solution became colorless, which was due to the consumption of IR783 and photobleaching of BODIPY.

### 2.6 IR783/BC NPs accumulated in tumors after systemic administration in tumor‐bearing mice

**FIGURE 6:**
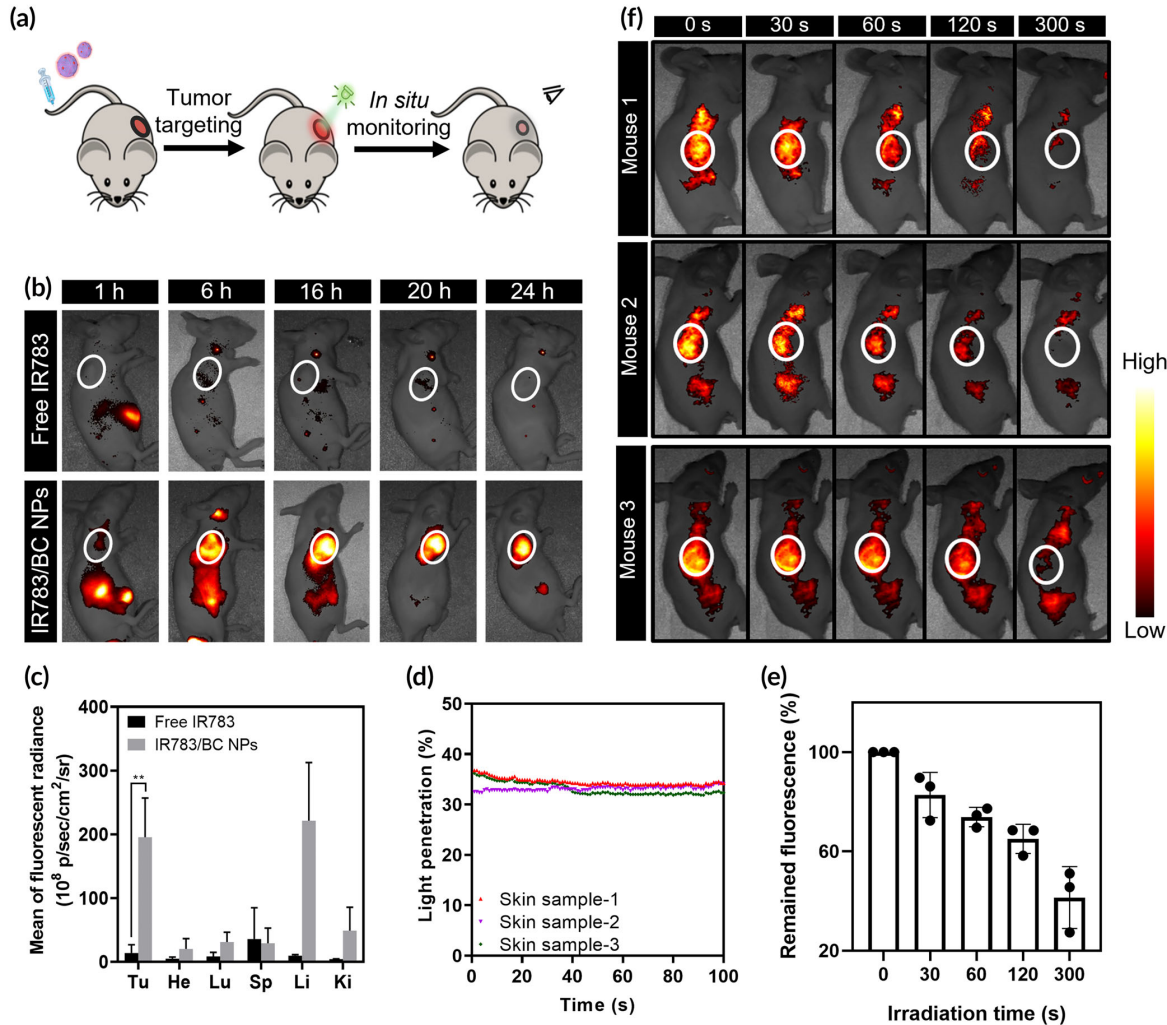
In vivo biodistribution and in‐situ monitoring upon light irradiation. (a) Schematic illustration of the treatment process in HCT116 tumor‐bearing mouse. (b) Representative IVIS fluorescent images of the mice post‐injection of free IR783 or IR783/BC NPs within 24 h (*n* = 3) (Ex. 780 nm). (c) Quantitative analysis of the biodistribution determined by IVIS in major organs and tumors. Tu, He, Lu, Sp, Li, and Ki represent tumor, heart, lung, spleen, liver, and kidney, respectively. **p < 0.01. (d) Quantitative analysis of the penetration efficiency of 530 nm green light through mouse skin samples (*n* = 3) within 100 s. (e) Quantitative analysis of fluorescence intensity in the tumor regions exposed to light irradiation for various durations. (f) In‐situ fluorescence imaging of the mice at 24 h post‐injection of IR783/BC NPs with light irradiation  at the tumor sites for various durations (*n* = 3). The tumor sites are marked by white circles. Light irradiation: 530 nm, 100 mW/cm^2^

As aforementioned, the sulfonate groups of the IR783 dye may attribute to the tumor‐targeting performance of IR783/BC NPs by CAV‐1‐mediated transcytosis. In addition, fluorescence imaging based on the emission changes of IR783 upon light irradiation could be expected to provide the possibility for in vivo monitoring of drug delivery (Figure 6a). The fluorescence imaging of HCT116 tumor‐bearing mice was recorded by in vivo imaging system (IVIS) at 1, 6, 16, 20, and 24 h after the intravenous injection of free IR783 or IR783/BC NPs into the mice (Figure 6b). The fluorescence of free IR783 was fleetly eliminated after the systemic administration, due to the fast removal of the free dye from the blood circulation. In contrast, IR783/BC NPs showed a longer retention post‐injection. The intensity of the NIR fluorescence in the tumor region gradually increased within 24 h after the injection of IR783/BC NPs, indicating that the nanoparticles possessed enhanced accumulation in the HCT116 tumors compared to the free dye, which can be explained by the combination of both the enhanced permeability and retention effect and the active targeting of IR783/BC NPs toward HCT116 tumors. In addition, the tumors and main organs were excised for ex vivo fluorescence imaging at 24 h after administration (Figure 6c; Figure S18). Compared to free IR783, the nanoparticles exhibited much better tumor retention capability. Besides, obviously preferential accumulation of the nanoparticles was also observed in the liver. It should be noted that we can selectively activate the nanoparticles in the tumors by light while those nanoparticles in the liver will not be activated by light, which may reduce side effects.

### 2.7 530 nm green light can penetrate through mouse skin tissues

Green light penetration through skin tissues was determined by ex vivo testing. Briefly, mouse skin samples (*n* = 3) were excised from three different mice. Quantitative penetration efficiency was calculated by recording the light intensity after getting through the skin (Figure 6d). The average penetration efficiency of 530 nm green light through mouse skin (*n* = 3) was calculated as 34.0%. Thus, after taking the light penetration and the requirement of BC photocleavage into consideration, the light irradiation parameters were set as 100 mW/cm^2^ and 10 min for in vivo anticancer experiments.

### 2.8 In vivo fluorescence imaging of IR783/BC NPs enabled the monitoring of drug delivery

The in‐situ monitoring of photoinduced nanoparticle dissociation was examined in HCT116 tumor‐bearing mice. As shown in Figure 6e,f, it was observed that all the three mice treated with IR783/BC NPs showed strong fluorescence at the tumor sites at 24 h post‐injection, displaying the accumulation of the nanoparticles. The 530 nm light irradiation (100 mW/cm^2^) was applied topically on the tumors for various durations from 0 to 300 s. The fluorescence images were recorded right after the irradiation was finished, with a cumulative irradiation duration of 0, 30, 60, 120, 300 s, separately. The results revealed that the fluorescence intensity in the tumors gradually decreased with the increase of the irradiation duration, which was consistent with the in vitro photoresponsive properties of IR783/BC NPs. Therefore, the in vivo fluorescence imaging enabled in‐situ monitoring of the drug delivery, including nanoparticle accumulation and light‐triggered nanoparticle dissociation. The in‐situ monitoring of such process may provide important guidance to indicate the in vivo distribution and the real‐time decomposition of the nanoparticles, which can be utilized to reduce the overdose of drug or light.

### 2.9 IR783/BC NPs plus light irradiation efficiently inhibited HCT116 tumor growth in vivo

**FIGURE 7:**
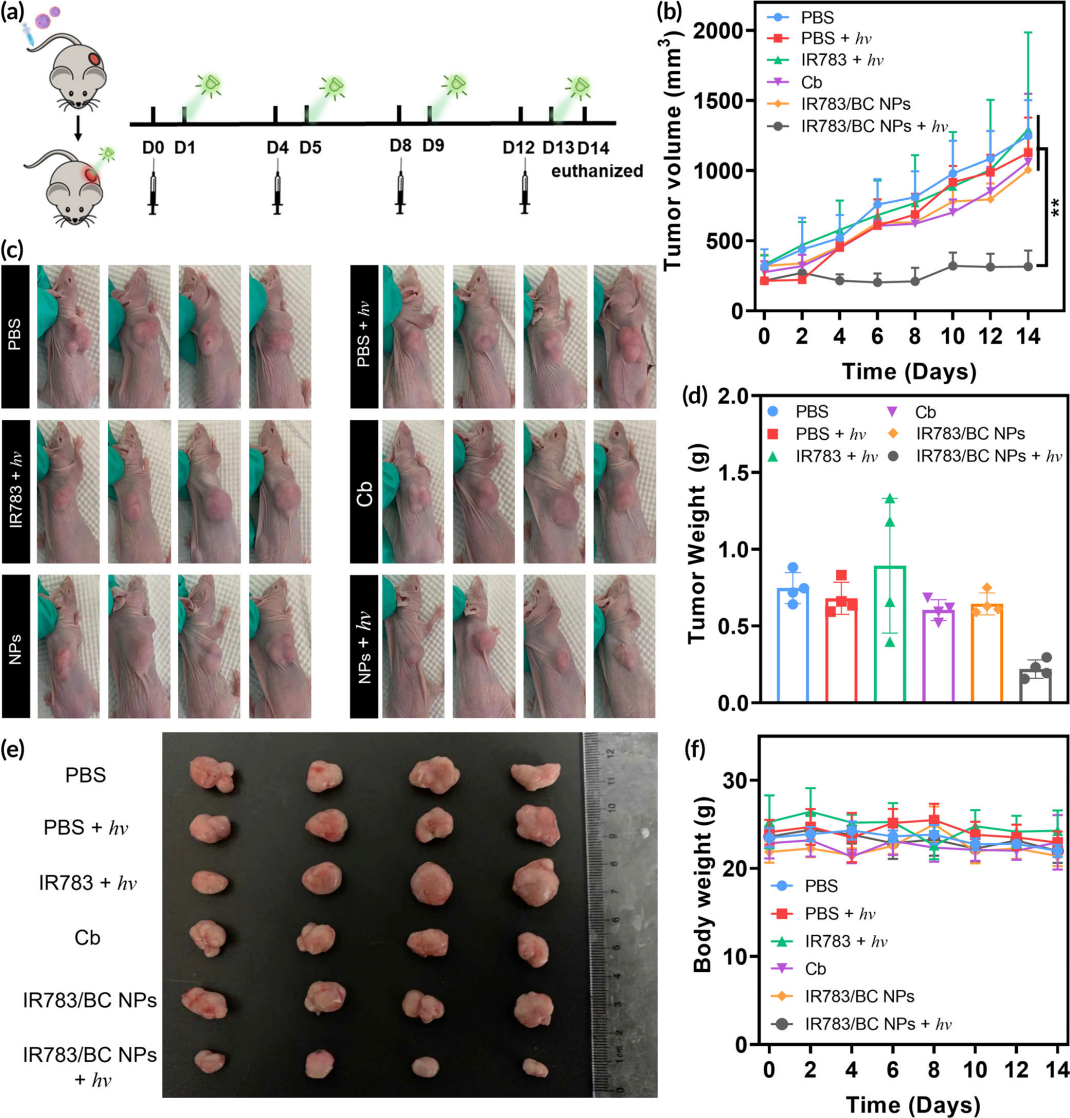
Antitumor efficacy in HCT116 tumor model. (a) Schematic illustrating treatment schedule for inhibiting subcutaneous tumor growth. (b) Tumor volume changes after treatments with PBS, IR783, Cb, IR783/BC NPs, and IR783/BC NPs with light irradiation, separately (*n* = 4). **p < 0.01. (c) Photos of the mice with subcutaneous tumors, (d) tumor weight, (e) photos of the tumors at Day 14 after different treatments as indicated. (f) Changes in body weight of the mice during the treatments. Light irradiation: 530 nm, 100 mW/cm^2^, 10 min

Encouraged by the in vitro cytotoxicity study and in vivo biodistribution study, we subsequently performed in vivo therapeutic efficacy study to illustrate the advantages of the photoresponsive IR783/BC NPs. BALB/c‐nude mice with HCT116 subcutaneous xenografts at around 200 mm^3^ were randomly divided into six groups (a. PBS; b. PBS + *hv*; c. IR783 + *hv*; d. free Cb; e. IR783/BC NPs; f. IR783/BC NPs + *hv*). The corresponding formulations were intravenously injected into the mice via tail vein. At 24 h post‐injection, 530 nm light irradiation (100 mW/cm^2^, 10 min) was applied topically onto the tumors (Figure 7a). The treatments were repeated every 4 days (on Day 0, 4, 8, and 12). As shown in Figure 7b,c, treatment with IR783/BC NPs plus light irradiation significantly suppressed the tumor growth while other groups only displayed limited therapeutic effects. It should be noted that the antitumor efficacy of free Cb was undermined in vivo, which may be due to its hydrolysis and fast clearance in blood.^46^ The nanoparticles without light irradiation also showed poor efficacy, indicating the therapeutic effect of IR783/BC NPs would only be activated upon light irradiation.

**FIGURE 8:**
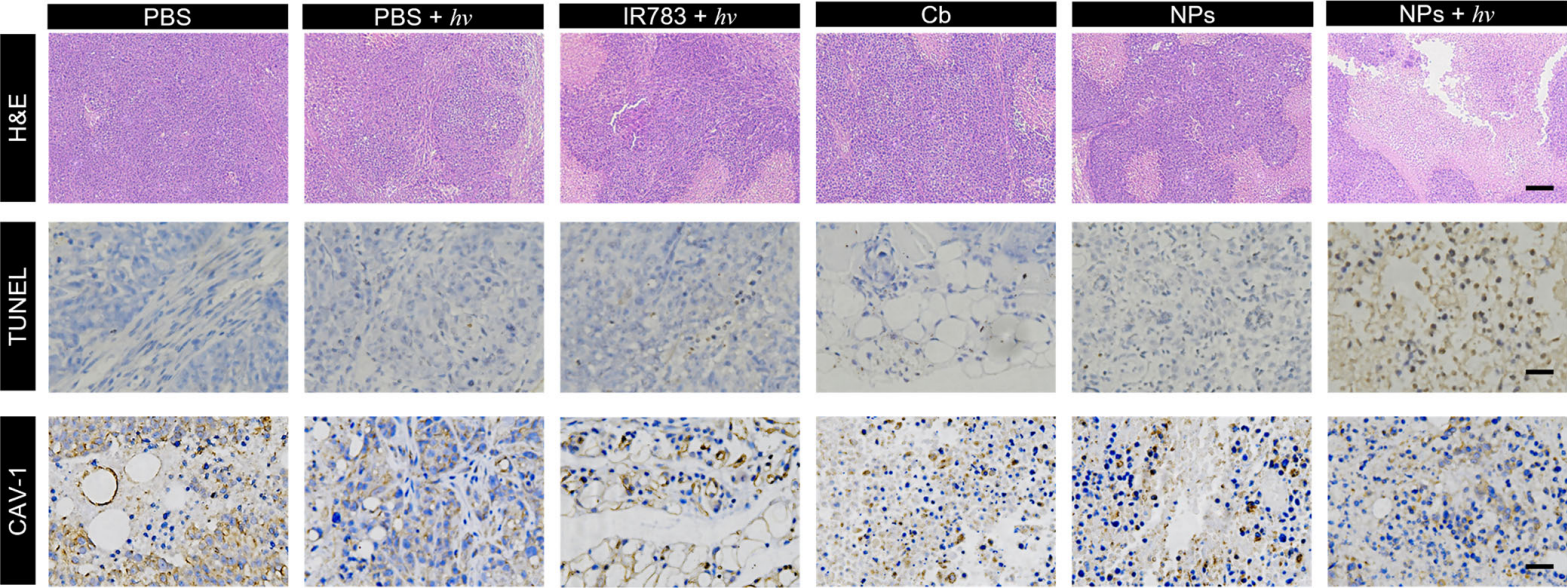
Representative H&E staining, TUNEL staining and immunobiological staining of CAV‐1 protein of the tumor sections. Scale bar: 200 μm (H&E), 50 μm (TUNEL), and 50 μm (CAV‐1). Light irradiation: 530 nm, 100 mW/cm^2^, 10 min

After 14‐day monitoring, the mice were sacrificed, and the tumors were resected for ex vivo characterizations. The tumor size and weight of the group treated with IR783/BC NPs plus light irradiation were significantly lower than other groups, which further supported the above results (Figure 7d,e). Additionally, no remarkable body weight loss was observed during the treatments with these formulations, suggesting good biocompatibility of the treatments (Figure7f). Moreover, the cell proliferation and apoptosis in tumors were analyzed by hematoxylin and eosin (H&E) staining and immunohistochemistry staining, respectively. The results shown in Figure 8 revealed that IR783/BC NPs with light irradiation effectively inhibited proliferation and induced apoptosis in the tumor tissues. As expected, immunohistochemistry staining of CAV‐1 also confirmed its high expression in all the tumors. As compared to the normal tissues (heart, liver, spleen, lung, and kidney), a larger area in the tumor tissue displayed CAV‐1 positive (brown areas in Figure S19). Moreover, based on the H&E staining results (Figure S20), the main organs exhibited no apparent necrosis or histological change after the treatments, suggesting minimal systemic toxicity. The secretion of some of the liver enzymes such as alanine aminotransferase and aspartate aminotransferase in the serum of the mice after different treatments was investigated and no obvious differences between all the groups were found (Figure S21). All these in vivo data suggested that the IR783/BC NPs could serve as a promising nanomedicine against tumors with light‐controllable activity, resulting in enhanced antitumor efficacy and biosafety.

## 3 CONCLUSIONS

Herein, we designed a simple yet novel strategy to fabricate photoresponsive nanomedicine by co‐assembly of photocleavable prodrug and IR783 dye. Interestingly, the incorporation of IR783 as the stabilizer enabled both high loading capacity of BC prodrug (~99%) and excellent stability of IR783/BC NPs. Upon 530 nm green‐light irradiation, IR783/BC NPs dissociated, released anti‐cancer agent Cb, and generated ^1^O_2_, exhibiting light‐triggered antitumor effect. The NIR fluorescence emission of IR783 in the nanoparticles displayed an “ON‐to‐OFF” pattern while applying light irradiation, which enabled in‐situ fluorescence imaging of light‐triggered dissociation of the nanoparticles. Additionally, the sulfonate groups of IR783 enabled the enhanced tumor accumulation of IR783/BC NPs by CAV‐1‐mediated transcytosis. Both the in vitro and in vivo studies verified the light‐triggered antitumor efficacy of IR783/BC NPs that integrated capabilities of tumor‐targeting, fluorescence monitoring, and light‐triggered therapeutic effect, resulting in effective tumor elimination in HCT116 tumor‐bearing mice.

Light‐responsive drug delivery systems have been shown to be useful for cancer treatment by applying light to trigger drug release and accumulation. The wavelength of light used for photoresponsive drug delivery is of great importance.^12^ The most frequently used light for the activation of the systems is UV (<400 nm) light, of which the tissue penetration is limited.^52^, ^53^ The longer wavelength light would allow deeper tissue penetration.^53^, ^54^ In this study, green light was used, which was reported to trigger the phototargeting of nanoparticles in a subcutaneous tumor model^40^ and trigger drug release from nanoparticles in the eye.^55^ The results of in‐situ fluorescence monitoring and tumor growth inhibition indicated that the 530 nm green light can reach the subcutaneous HCT116 tumors for light‐triggered cancer therapy. Therefore, such nanomedicine may be applied for drug delivery to superficial or easily accessible tissues, like skin or eyes. For diseased lesions deep in the body, light delivery by optical fibers may be an option.^56^, ^57^

IR783 presented versatile functions by serving as a stabilizer, tumor‐targeting moiety, and imaging agent in our system. Though IR783 was widely used for imaging in clinic,^58^ its application in drug delivery has just taken its first step. Some nanosystems that involved IR783 as a stabilizer have been reported recently.^31^, ^42^ Nevertheless, for the first time, this work demonstrated IR783 as a “three‐birds‐with‐one‐stone” agent in a simple prodrug‐dye co‐assembled nanoparticle, especially as a reporter for in‐situ monitoring of nanoparticle dissociation and drug release. IR783 exhibits such functions because of its integration in the photoresponsive system, for example, the photoresponsive prodrug‐dye nanoparticles in this study.

In summary, a simple and reliable co‐assembly strategy for developing photocleavable prodrug‐dye co‐assembled nanomedicine was reported. It is a promising platform for precisely and remotely controllable cancer therapy. The study may open a new path for fabricating multifunctional photoresponsive drug delivery systems with ultrahigh loading capacity in a simple, reliable, and clinically transferable way. Other photoresponsive prodrugs, especially long‐wavelength light‐excitable prodrugs, are worthy of being explored to form similar nanomedicines for specific disease treatment.

## 4 MATERIALS AND METHODS

### 4.1 Materials and instruments

Indocyanine IR783 dye was purchased from Tokyo Chemical Industry (TCI) Co., Ltd. Chlorambucil (Cb) was purchased from J&K Scientific. Dimethyl sulfoxide (DMSO) was purchased from DUKSAN Pure Chemicals. FBS, RMPI 1640, MTT, penicillin–streptomycin were purchased from Gibco. PBS was purchased from Sigma‐Aldrich. All chemicals were used directly without further purification. Deionized water was used to prepare all aqueous solutions. The UV‐Vis absorption spectra and cell viability were measured by a multi‐mode microplate reader (SpectraMax® M4, Molecular Devices LLC) using quartz cuvettes with 1‐cm path length (Starna Scientific Ltd.). Size and zeta potential of nanoparticles were measured by Zetasizer Nano ZS90 (Malvern Instrument). TEM images were obtained by CM100 Transmission Electron Microscope (Philips). The products after photolysis were quantitatively determined by 1260 Infinity II HPLC (Agilent Technologies). The excitation light source for photolysis and cell study was green‐light LED (530 nm) (Mightex). The excitation light source for in vivo study was green‐light laser (530 nm) (Laserwave Co. Ltd.). The light irradiance was determined by PM100 USB power and energy meter (Thorlabs) with S142C integrating sphere photodiode power sensor (Si 350–1100 nm, Thorlabs).

### 4.2 Screening of different concentrations of IR783 for co‐assembly

BODIPY‐Cb (20 μL, 10 mg/mL, in DMSO) was added dropwise (20 μL per 10 s) to a group of 300 μL aqueous solutions containing gradient concentrations of IR783 (10, 50, 100, 200, 400, and 800 μg/mL) under vigorous vortex. Then the particle size and PDI of each solution were measured by DLS.

### 4.3 Preparation of IR783/BC NPs

BODIPY‐Cb was dissolved in DMSO (20 μL, 10 mg/mL), and added dropwise to a 300 μL aqueous solution containing IR783 (400 μg/mL) under vigorous vortex. The solution was centrifuged once (2000 × *g*, 10 min, 4 °C) to remove the precipitate. Then the supernatant was centrifuged twice (30,000 × *g*, 30 min, 4 °C), and the resulting precipitate was re‐suspended in 300 μL of sterile PBS. The contents of IR783 and BC in the resulted nanoparticle solution were determined by HPLC analysis. Their loading capacity and encapsulation efficiency were calculated as follows:

$$\text{Loading capacity}\;\left(\%\right)=\frac{\text{Weight of loaded}\;\mathrm{IR}783\;\text{or}\;\mathrm{BC}}{\text{Weight of loaded nanoparticles}\;}\times 100\%$$

$$\text{Encapsulation efficiency}\;\left(\%\right)=\frac{\text{Weight of loaded}\;\mathrm{IR}783\;\text{or}\;\mathrm{BC}}{\text{Weight of}\;\mathrm{fed}\;\mathrm{IR}783\;\text{or}\;\mathrm{BC}\;}\times 100\%$$

### 4.4 Stability of IR783/BC NPs

IR783/BC NPs (50 μM) were re‐suspended in water, PBS, RPMI 1640 cell culture medium, or FBS‐containing DMEM medium at 37 °C in plastic cuvette shielded from light. The particle size and PDI were measured every 2 h by DLS within a total period of 48 h.

### 4.5 Photocleavage of prodrug in IR783/BC NPs

500 μL of the nanoparticle solution (50 μM) was irradiated by the LED light (530 nm, 50 mW/cm^2^) for different time periods (1, 2, 3, 5, 7, and 10 min). The irradiance of the LED irradiation was measured with a power and energy meter. After the irradiation, the size, morphology, and content of the solutions were analyzed by DLS, TEM, and HPLC, respectively. To quantitatively determine the uncleaved BODIPY‐Cb and released Cb, the aqueous solution of IR783/BC NPs (100 μM, on the basis of BC prodrug) was exposed to 530 nm laser (50 mW/cm^2^) for different time periods (1, 3, 5, 7, and 10 min). The irradiated samples (50 μL) were mixed with 50 μL acetonitrile to dissociate the assemblies and analyzed by HPLC. For HPLC analysis, the injection volume for all samples was 50 μL, and the detection wavelength was 260, 540, and 783 nm. Acetonitrile/water with 0.1% trifluoroacetic acid was chose as the mobile phase, while the following gradient of acetonitrile and water was selected to achieve separation of different components.

**Table:** 

Time (min)	Acetonitrile (%)	Water (%)
0	20	80
5	20	80
30	95	5
35	95	5

The flow rate of the mobile phase was 1.5 mL/min. Experiments under each set of conditions were repeated for at least three times.

### 4.6 Spectrum study of IR783 under light irradiation

The degradation of IR783 was demonstrated by spectroscopy. The IR783/BC NPs solution was irradiated for different time periods (530 nm, 50 mW/cm^2^, 0–60 s). At different time points, the absorbance and fluorescence were measured by spectrometers without any dilution.

### 4.7 Cell culture

HCT116 cells were cultured in RPMI‐1640 (Gibco) supplemented with 10% FBS (Gibco) and 100 units/mL antibiotics (Penicillin–Streptomycin, Gibco) at 37 °C in a 5% CO_2_ humidified atmosphere. HUVECs cells were cultured in DMEM (Gibco) supplemented with 10% FBS (Gibco) and 100 units/mL antibiotics (Penicillin–Streptomycin, Gibco) at 37 °C in a 5% CO_2_ humidified atmosphere.

### 4.8 Singlet oxygen detection

Singlet oxygen generation was measured both in solutions and cells using SOSG and DCFH‐DA as indicator, respectively. Briefly, free IR783, free BC, and IR783/BC NPs (at the equivalent concentration of BC at 1 μM) was mixed with SOSG probe (5 μM) in PBS. The solutions were exposed to light irradiation and the fluorescence intensity was detected at predetermined time intervals (Ex. 488 nm). Besides, the intracellular ^1^O_2_ generation was detected by CLSM. HCT116 cells were seeded in confocal dishes (Corning) at a density of 10,000 cells/well and treated with different formulations for 6 h. Subsequently, the cells were washed with PBS for three times and incubated with DCFH‐DA (10 μM) for 30 min. The cells were irradiated by LED (530 nm, 50 mW/cm^2^, 5 min) for the light irradiation groups. The intracellular fluorescence was observed by CLSM imaging and flow cytometry to evaluate the ^1^O_2_ generation.

### 4.9 Cellular uptake study

Cellular uptake of free IR783 or IR783/BC NPs was investigated by using CLSM imaging. In brief, HCT116 cells were seeded in confocal dishes at a density of 10,000 cells/well and treated with different formulations for 6 h in the dark. The cells were washed by PBS for three times and stained with Hoechst 33342. Then the cells were directly observed by CLSM imaging and analysized by flow cytometry.

HCT116 cells were treated with IR783/BC NPs (10 μM) in the dark for 4 h. For the 24‐h group, the nanoparticles‐containing medium was replaced by fresh medium after 4 h and the cells were incubated for another 20 h. For the light irradiation group, green‐light irradiation (50 mW/cm^2^, 10 min) was performed after 2 h incubation, and then the cells were incubated for another 2 h (the total incubation time was 4 h). After staining with LysoTracker® Green DND‐26 (Thermo Fisher), cells were washed with PBS for three times and imaged by a confocal microscope (Zeiss, LSM 980) (Ex. 488, 639 nm).

Endocytosis inhibitors (chlorpromazine, genistein and mβ‐CD; all from Sigma‐Aldrich) were dissolved in DMSO as stock solutions. HCT116 cells were incubated at 37 °C for 1 h in complete medium with chlorpromazine (5 μg/mL), genistein (25 μg/mL), or mβ‐CD (5 μg/mL). The cells were washed with PBS and then incubated with 10 μM IR783/BC NPs for 2 h. Following this, the cells were washed and collected for further analysis.

### 4.10 Cytotoxicty study

The cell viability was evaluated by MTT assay. HCT116 cells were incubated in 96‐well microtiter plates for 24 h. Then, gradient concentrations of BC prodrug or IR783/BC NPs in RPMI 1640 culture medium were added to the cells. After 6 h incubation, the residual BC or IR783/BC NPs were removed by fresh RPMI 1640 culture medium (100 μL). Then, the cells were irradiated by 530 nm LED light (50 mW/cm^2^, 5 min) or incubated at room temperature without light irradiation. After another incubation for 24 h, MTT solution (10 μL, 10 mg/mL) was added to the cells to cultivate for 3 h. Next, the cell culture medium was discarded, and DMSO (100 μL) was added. The absorbance was measured by a multi‐mode microplate reader at 490, 570, and 630 nm.

### 4.11 Live/Dead staining and apoptosis detection

For live/dead staining, HCT116 cells were seeded in confocal dishes at a density of 10,000 cells/dish and treated with PBS, free IR783, free BC and IR783/BC NPs, separately, with an equivalent concentration of BC at 50 μM. For the irradiation groups, cells were washed with PBS for three times after 6 h. And then fresh medium was added into the dish and 530 nm LED irradiation was applied for 5 min. Calcein‐AM and PI were then added into the medium and the cells were observed by CLSM without any treatment.

For apoptosis study, HCT116 cells were seeded in 6‐well plates at a density of 50,000 cells/well and treated with free Cb or IR783/BC NPs at the equivalent concentration of Cb at 5 and 20 μM for 6 h. Then the cells were washed by PBS. The cells in the irradiation group were irradiated by 530 nm LED for 5 min. After 24‐h incubation, the cells were washed with PBS for three times, collected by trypsin digestion, and stained with Annexin‐V/FITC apoptosis kit. ACEA NovoCyte Quanteon flow cytometer (ACEA Biosciences) was used for apoptosis analysis.

### 4.12 Animal study

All the animal experiments were performed in compliance with the Guidelines of the Care and Use of Laboratory Animals of Fudan University and approved by the Animal Ethics Committee of Fudan University. BALB/c nude mice were purchased from SLAC Experiment Animal Co., Ltd. and kept in the Laboratory Animal Center of Fudan University. For the subcutaneous tumor model, male BALB/c nude mice were injected with 2 × 10^7^ HCT116 cells subcutaneously. The mice were then further kept in SPF condition for 5–7 days until tumors were observed.

### 4.13 In vivo biodistribution

The biodistribution of IR783/BC NPs in the HCT116 tumor model was visualized by an in vivo fluorescence imaging system (Cailper PerkinElemer). IR783, as a NIR fluorescent dye, was directly detected for living imaging. Mice were treated with free IR783 or IR783/BC NPs via intravenous injection with a dose of IR783 at 100 μg/kg. Fluorescence imaging was performed at 1, 6, 16, 20, and 24 h post‐injection. At 24 h, the mice were euthanized and tumors and major organs (heart, liver, spleen, lung, and kidney) were excised for ex vivo imaging.

### 4.14 In‐situ monitoring of light‐triggered therapy

HCT116 tumor‐bearing mice were intravenously injected with IR783/BC NPs with a dose of IR783 at 100 μg/kg. The mice were further kept in the dark for 24 h, and light irradiation (530 nm, 100 mW/cm^2^) was applied on the tumor sites. Fluorescence imaging was performed before the irradiation and at 30, 60, 120, and 300 s post‐irradiation. The images and fluorescent intensity were recorded for analysis.

### 4.15 In vivo antitumor efficacy

The antitumor efficacy of IR783/BC NPs in the presence or absence of light was investigated in HCT116 tumor mouse model. The mice were randomly divided into six groups when the tumor size reached about 200 mm^3^. Different groups of mice were treated with different formulations via intravenous injection at Day 0, 4, 8, and 12: (1) PBS; (2) PBS + *hv*; (3) IR783 + *hv*; (4) Cb; (5) IR783/BC NPs; (6) IR783/BC NPs + *hv*. For the irradiation groups, light irradiation (100 mW/cm^2^, 10 min) was performed at 24 h post‐injection. The dose for each formulation was set as 5 mg/kg Cb equivalent. Tumor size and body weight of the mice were measured in a 2‐day interval, and the tumor volume was calculated as V = 1/2 × width^2^ × length. At the end of the experiment (Day 14), all mice were euthanized, and the tumors and major organs were collected and sliced for H&E staining and immunohistochemical analysis.

### 4.16 Statistical analysis

All experiments were conducted three times or more independently (*n* ≥ 3). Data were presented as the mean ± standard deviation (SD). The one‐way ANOVA‐LSD and Independent Samples *t*‐test were adopted to determine the statistical significance of differences by Graphpad Prism 8.0 software.

## AUTHOR CONTRIBUTIONS

**Kaiqi Long:** Data curation (lead); investigation (equal); methodology (equal); writing – original draft (lead); writing – review and editing (lead). **Yifan Wang:** Investigation (lead); methodology (equal); writing – original draft (equal). **Wen Lv:** Investigation (equal); methodology (equal). **Yang Yang:** Methodology (equal); writing – review and editing (equal). **Shuting Xu:** Writing – review and editing (equal). **Changyou Zhan:** Methodology (equal); supervision (equal); writing – review and editing (supporting). **Weiping Wang:** Conceptualization (lead); funding acquisition (lead); methodology (equal); project administration (lead); supervision (lead); writing – review and editing (lead).

## CONFLICT OF INTERESTS

A US provisional patent application was filed with No. 63/262,789.

## Supporting information


**Appendix S1**: Supporting Information

## Data Availability

The data that support the findings of this study are available from the corresponding author upon reasonable request.
