# Supplementary material for: Photoresponsive prodrug‐dye nanoassembly for in‐situ monitorable cancer therapy
Source: Bioeng Transl Med. 2022 Mar 12;7(3):e10311. doi: 10.1002/btm2.10311 (PMC9472000; doi:10.1002/btm2.10311)
Supplement: Supplementary file 1 — Appendix S1: Supporting Information [file BTM2-7-e10311-s001.docx]

Supporting Information

Photoresponsive prodrug-dye nanoassembly for *in-situ* monitorable cancer therapy

*Kaiqi Long ^1,2,3^, Yifan Wang ^1,2,3^, Wen Lv ^1,2,3^, Yang Yang ^4,5^, Shuting Xu ^1,2,3^, Changyou Zhan ^4,5^, Weiping Wang ^1,2,3^**

1. State Key Laboratory of Pharmaceutical Biotechnology, The University of Hong Kong, Hong Kong, China.
2. Department of Pharmacology and Pharmacy, Li Ka Shing Faculty of Medicine, The University of Hong Kong, Hong Kong, China.
3. Laboratory of Molecular Engineering and Nanomedicine, Dr. Li Dak-Sum Research Centre, The University of Hong Kong, Hong Kong, China.
4. Department of Pharmacology, School of Basic Medical Sciences & State Key Laboratory of Molecular Engineering of Polymers, Fudan University, Shanghai, China.
5. School of Pharmacy, Fudan University & Key Laboratory of Smart Drug Delivery, Ministry of Education, Fudan University, Shanghai, China.

Corresponding Author:

Dr. Weiping Wang, E-mail: wangwp@hku.hk

**Contents**

1. Figure S1-S21...................................................................................................p3-15
2. Table S1............................................................................................................p4

**Figure S1**. Size and PDI of IR783/BC NPs when prepared with different concentrations of IR783 solutions (n = 3).


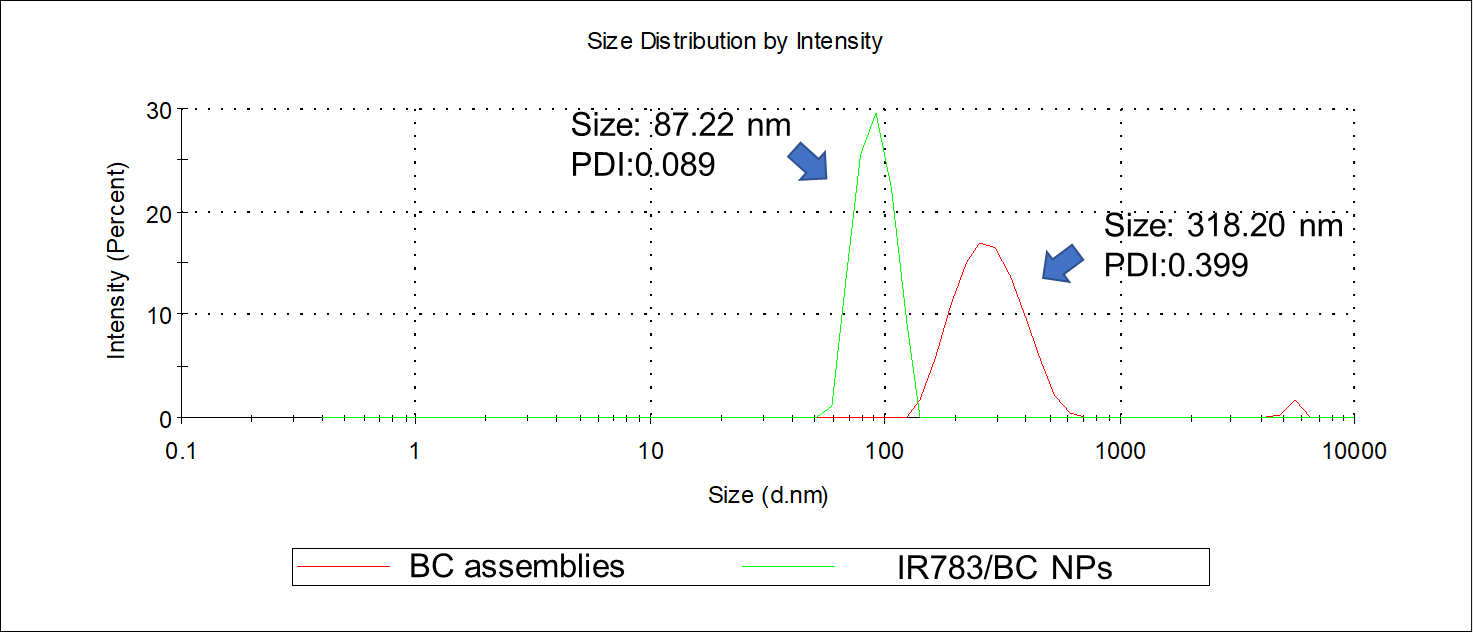


**Figure S2.** Size distribution of IR783/BC NPs and the BC assemblies while prepared *via* the same flash nanoprecipitation method.

**Table S1.** Loading capacity and encapsulation efficiency of IR783 and BC in IR783/BC NPs.


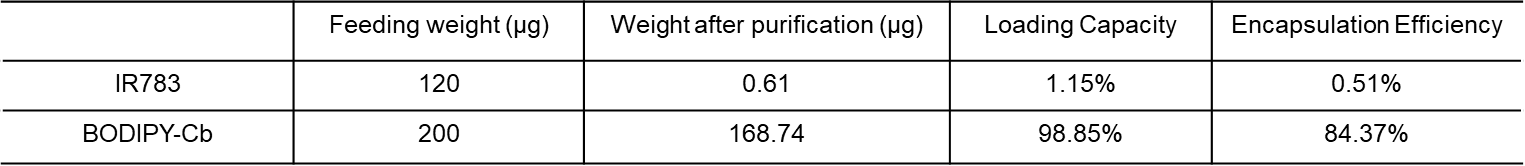


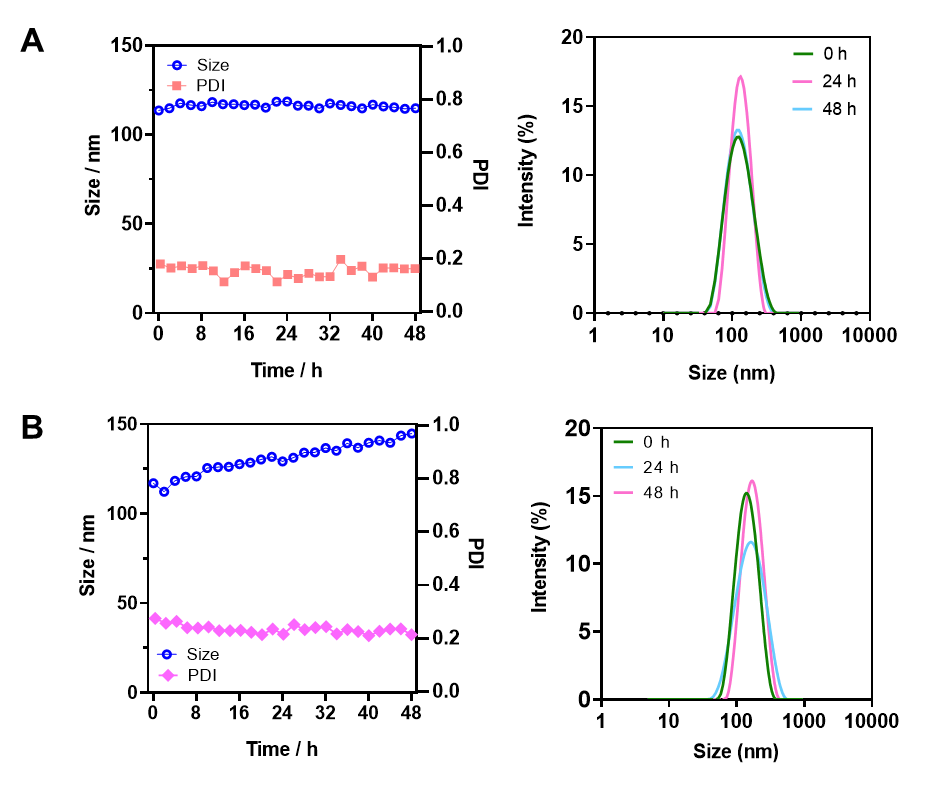


**Figure S3**. Stability and size distributions of IR783/BC NPs in (A) DMEM medium and (B) 10% FBS-containing DMEM medium at 37 ^o^C for 48 h.

**Figure S4.** Stability of IR783/BC NPs in water at 37 ^o^C for 48 h.


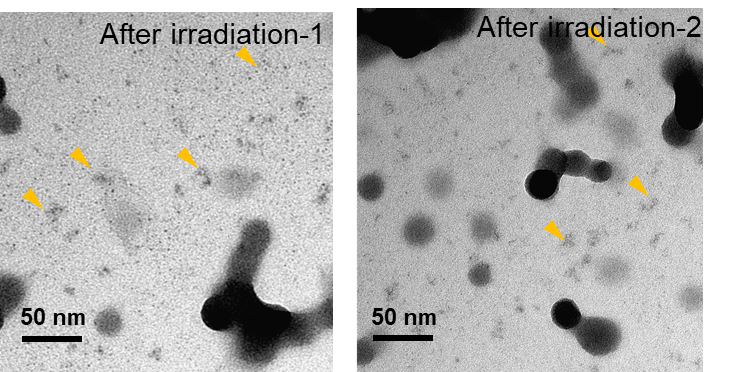


**Figure S5.** TEM images of IR783/BC NPs after light irradiation. The small fragments are pointed out with yellow arrows.

**Figure S6.** Precentages of remained Cb in water after incubation for different time periods at 37 ^o^C (mornitored by HPLC at 260 nm).


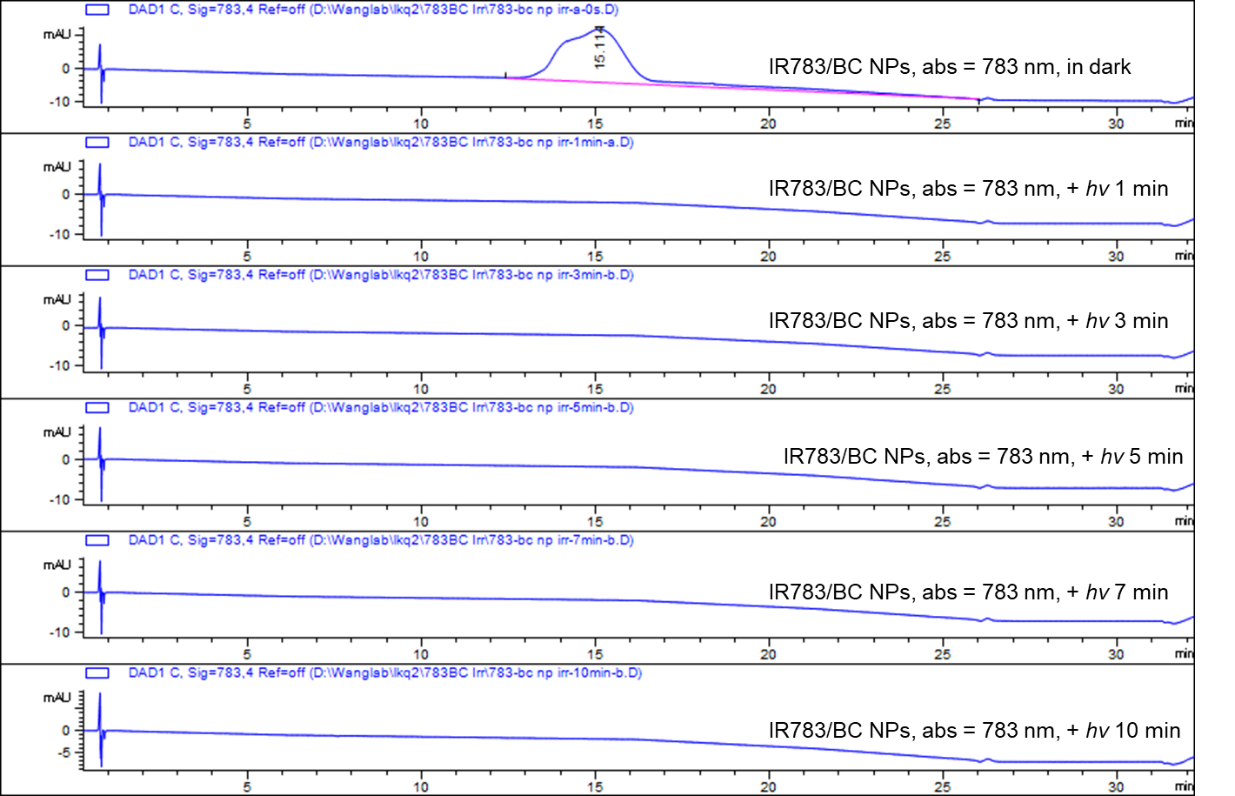


**Figure S7.** HPLC traces (detected at 783 nm) of IR783/BC NPs after different time periods (0, 1, 3, 5, 7 and 10 min) of light irradiation (530 nm, 50 mW/cm^2^).


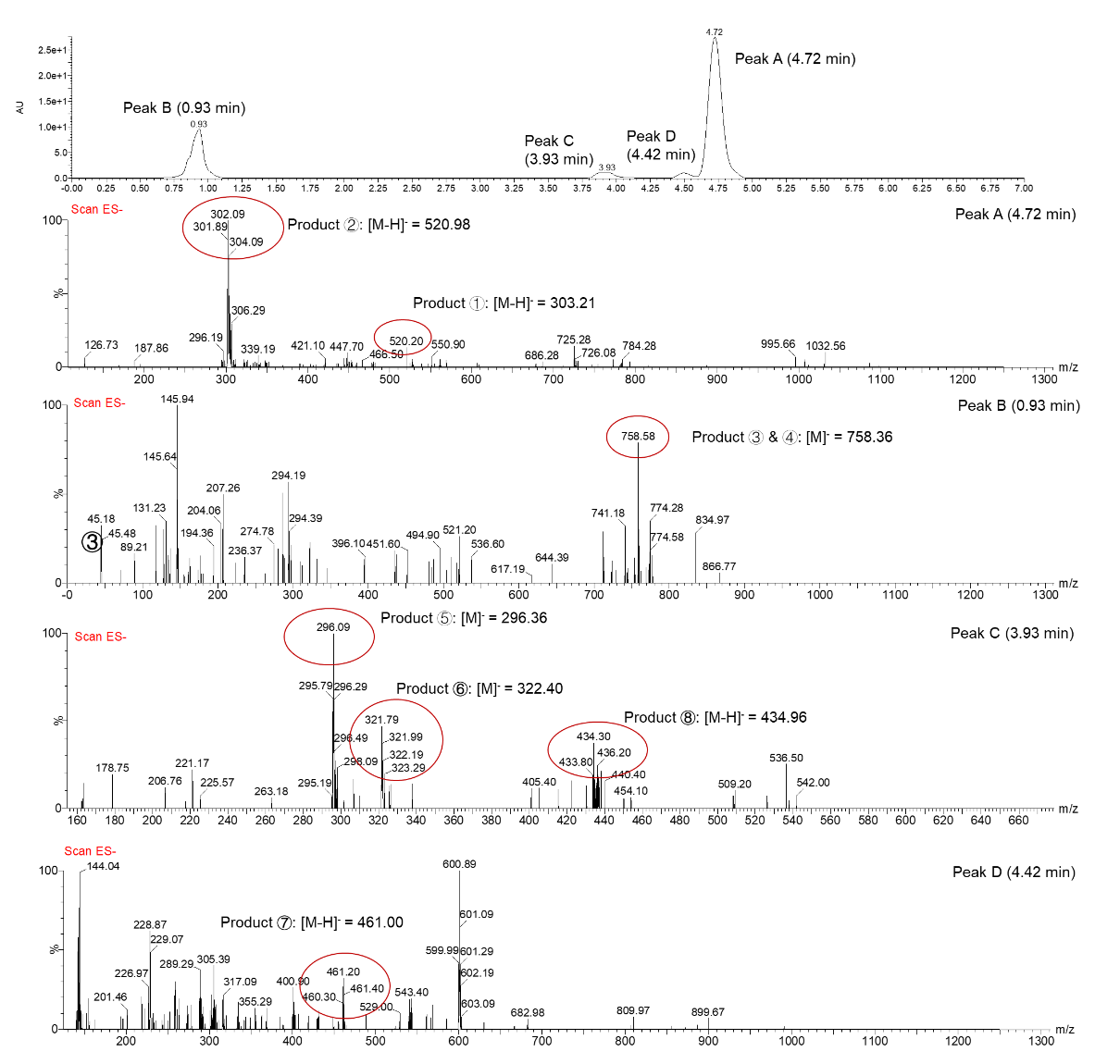


**Figure S8.** LC-MS spectra of the light-irradiated (530 nm, 50 mW/cm^2^) IR783/BC NPs solution.


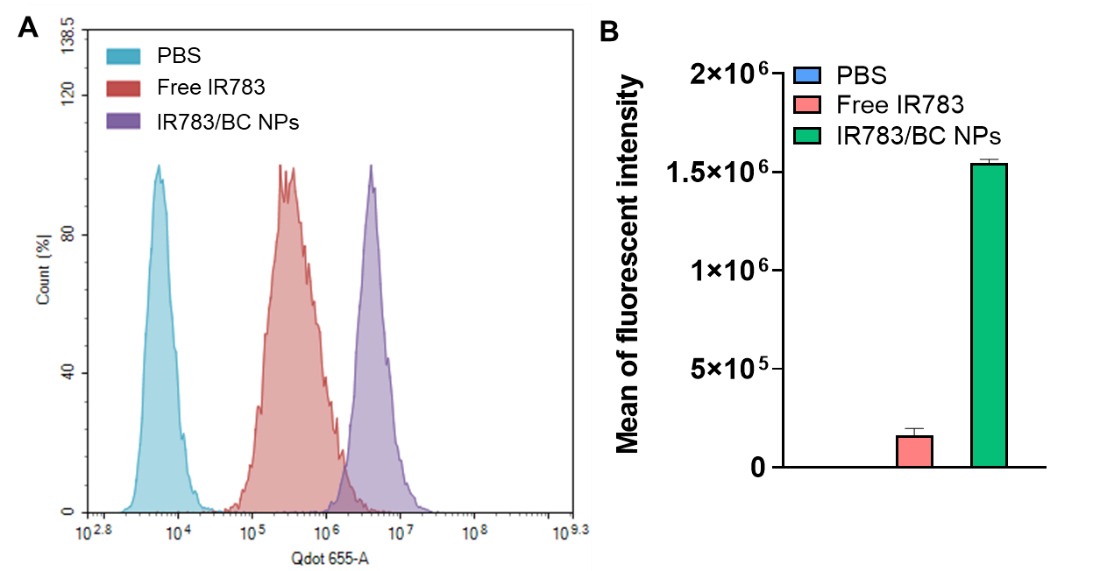


**Figure S9.** Flow cytometry analysis of intracellular uptake of IR783/BC NPs in HCT116 cells after 6-h incubation at 37 ^o^C.


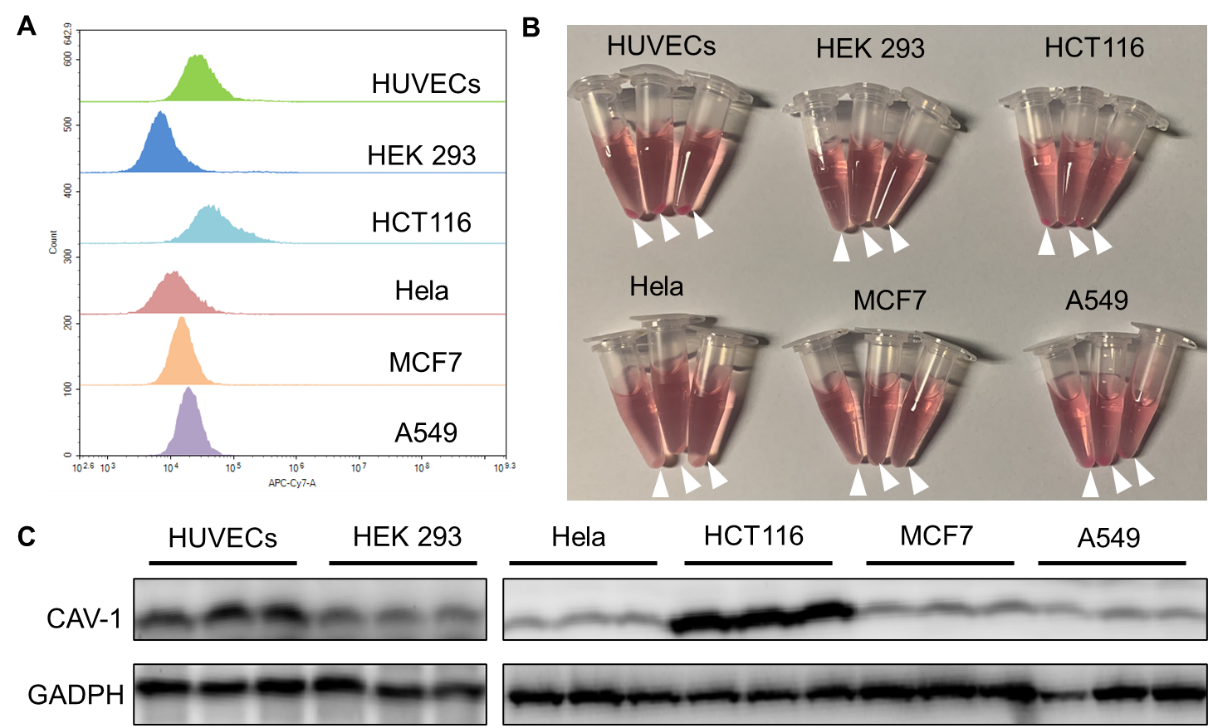


**Figure S10.** (A) Flow cytometry anlaysis of different cells incubated with IR783/BC NPs (10 μM) for 6 h in the dark at 37 ^o^C. (B) A photo of the cell pellets collected by centrifugation, displaying different shades of purple-red color. The result indicates different cellular uptake of the nanoparticles. (C) Western blotting of CAV-1 and GADPH in different cell lines.

**Figure S11.** Intracellular uptake of IR783/BC NPs in HCT116 cells incubated with various endocytosis inhibitors or incubated at 4 ^o^C.


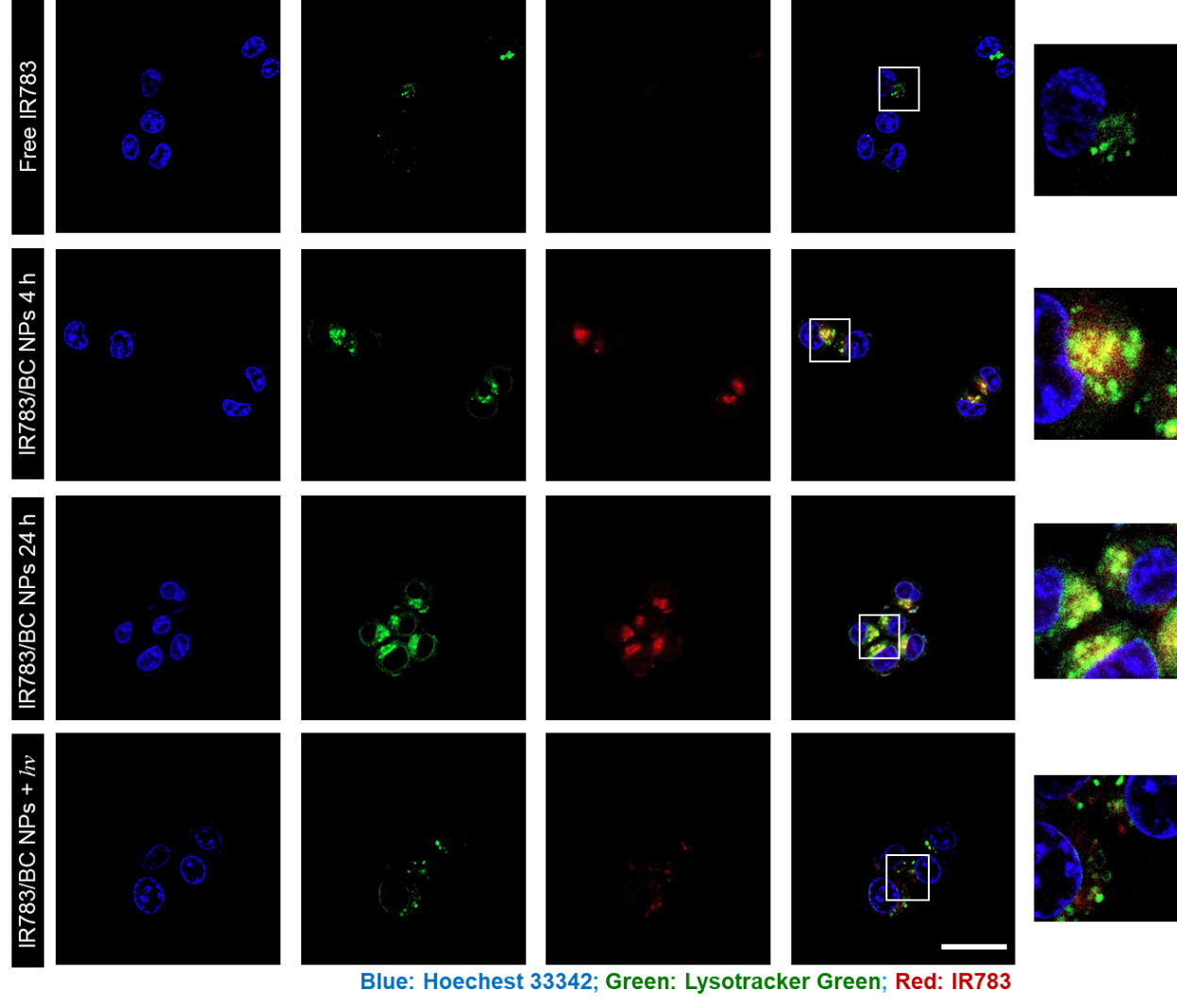


**Figure S12.** CLSM images of cells incubated with IR783/BC NPs for 4 h and 24 h, or upon 10-min green light irradiation (530 nm, 50 mW/cm^2^). Lysosomes were labelled with Lysotracker® Green. Scale bar: 20 μm.


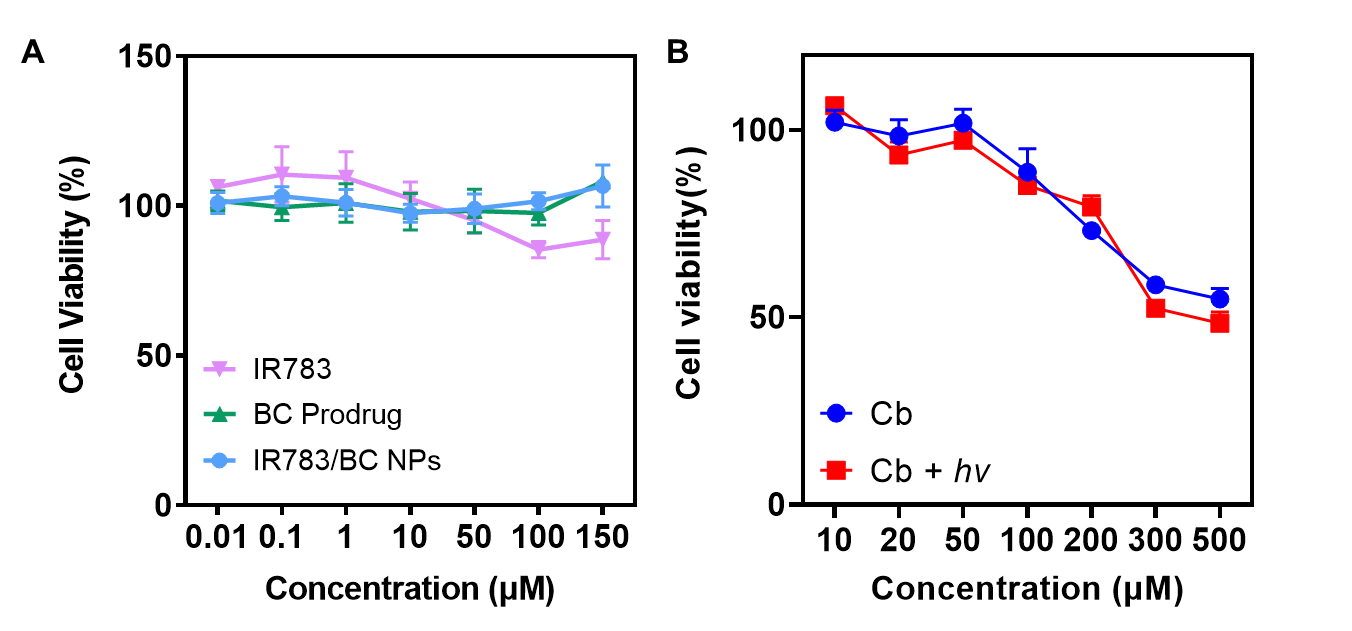


**Figure S13**. (A) Cell viability of HUVECs treated with IR783, BC and IR783/BC NPs in the dark. (B) Cytotoxicity of Cb against HCT116 cells with/without light irradiation (530 nm, 50 mW/cm^2^, 10 min).


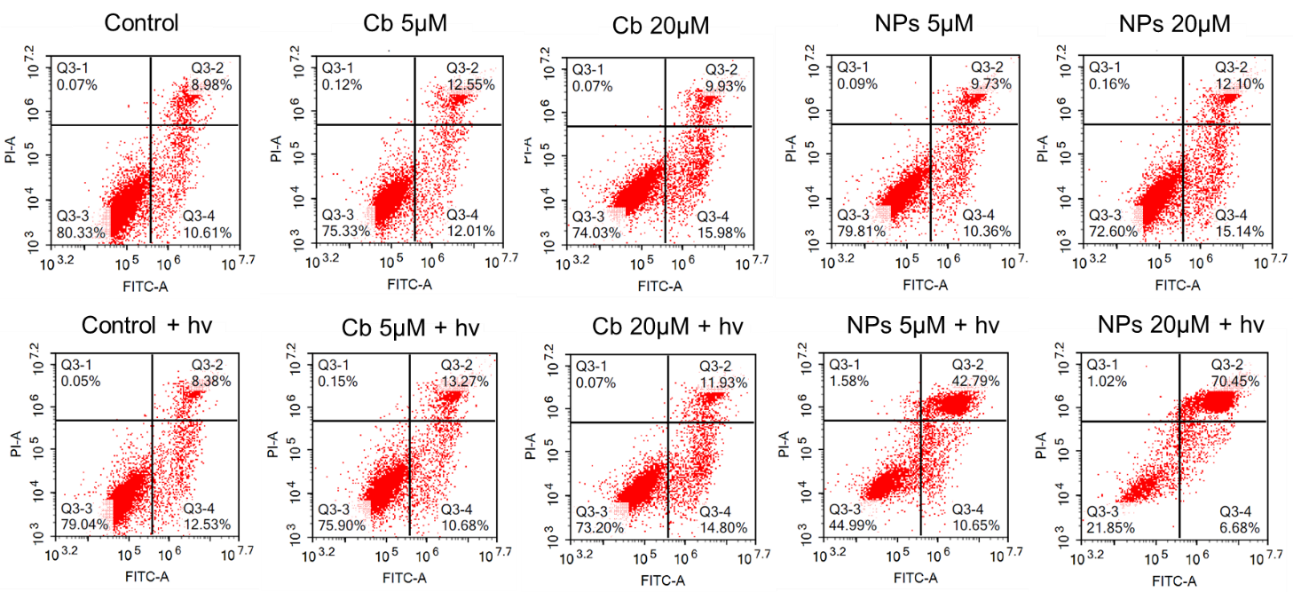


**Figure S14**. Apoptosis study of HCT116 cells treated with Cb and IR783/BC NPs with/without light irradiation (530 nm, 50 mW/cm^2^, 10 min).

**Figure S15.** UV-Vis absorption spectrum of IR783 (1 μM) in H_2_O.


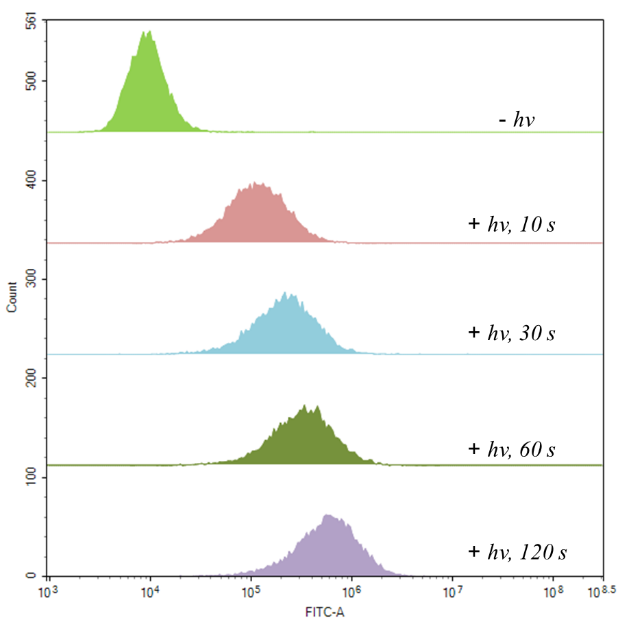


**Figure S16.** Flow cytometric quantification of the intracellular ROS generation of HCT116 cells incubated with IR783/BC NPs (5 μM) for 2 h and DCFH-DA (10 μM) for 30 min. Light irradiation (530 nm, 50 mW/cm^2^) was conducted after removing the nanoparticles-containing medium and the cells were collected for analysis.


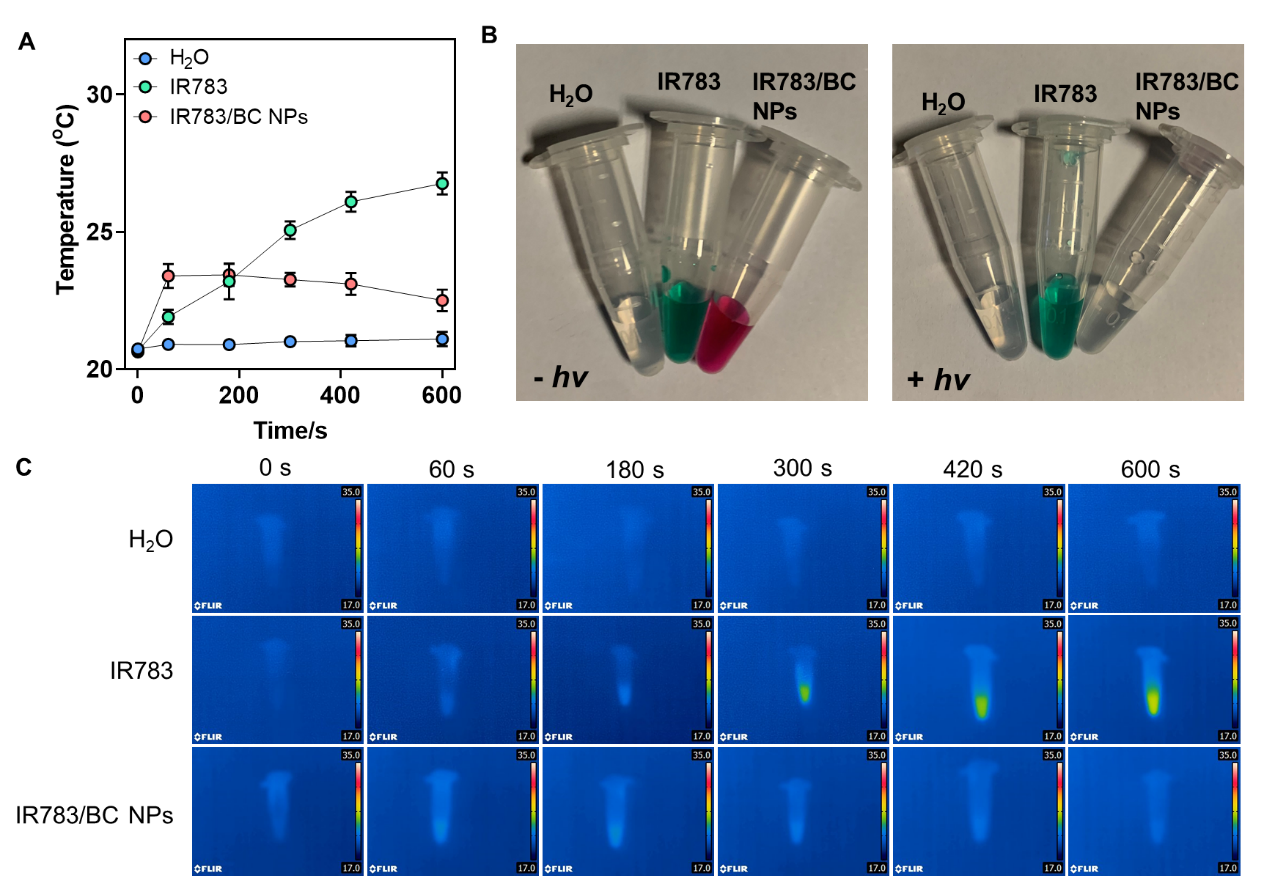


**Figure S17.** Photothermal effect of water, free IR783 and IR783/BC NPs (10 μM) under 530 nm light irradiation (50 mW/cm^2^) for different time periods. (A) Temperature change curves during the light irradiation. (n = 3) (B) Photos of the solutions before and after the light irradiation. (C) Thermal images of the solutions after the light irradiation for different time periods.


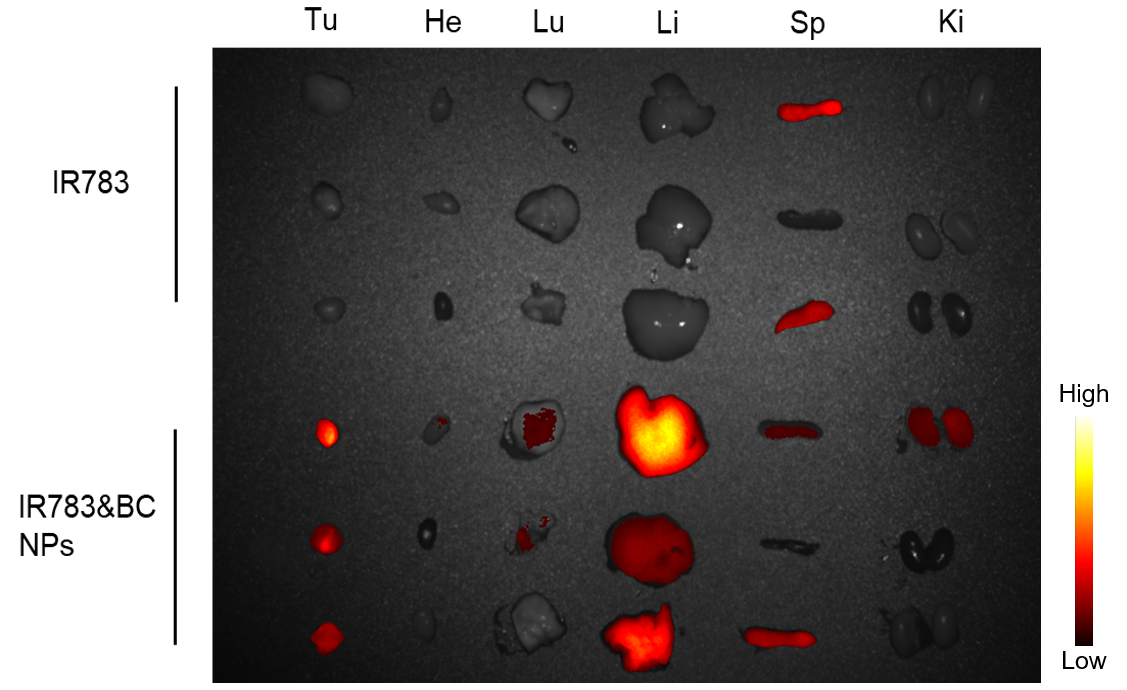


**Figure S18**. Representative fluorescent images of tumors and major organs at 24 h after the injection of IR783 and IR783/BC NPs, respectively.


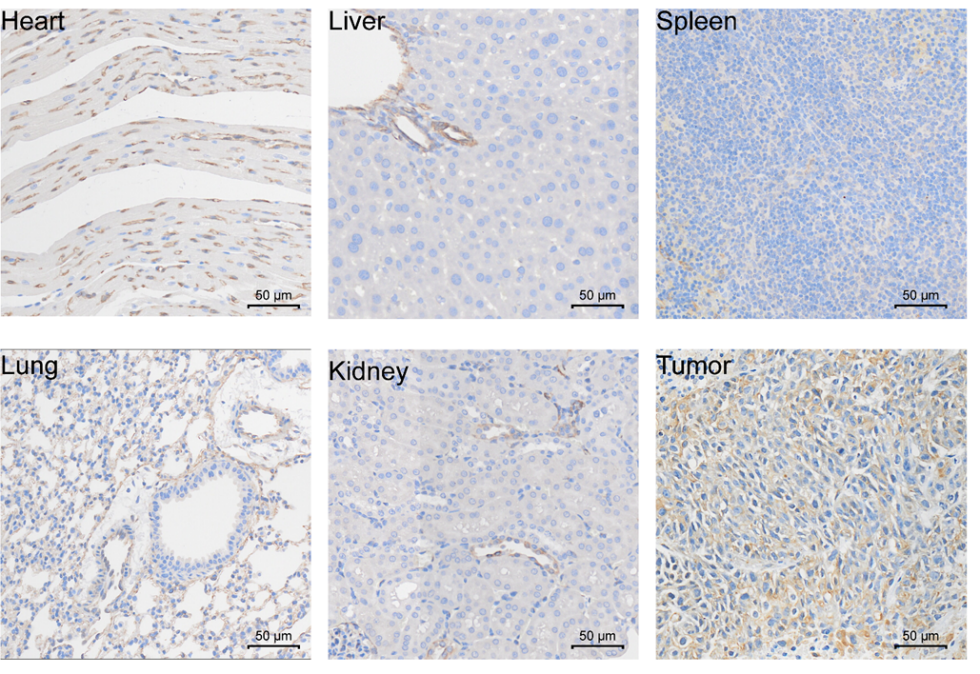


**Figure S19.** Immunobiological staining of CAV-1 protein in the heart, liver, spleen, lung, kidney and tumor tissues of the HCT116 tumor-bearing mice.


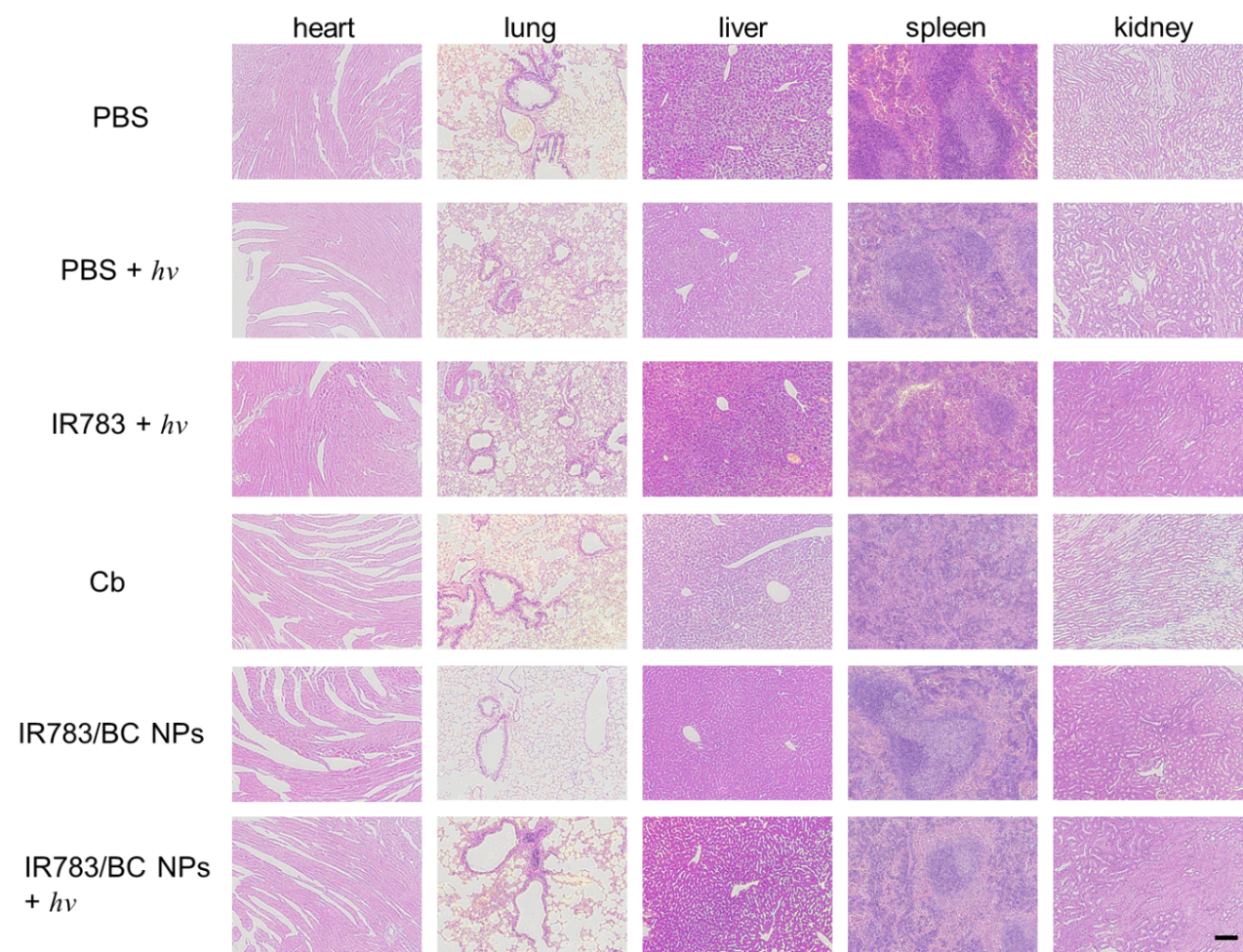


**Figure S20**. H&E staining of sections of major organs in the mice after different treatments. Scare bar: 200 μm.


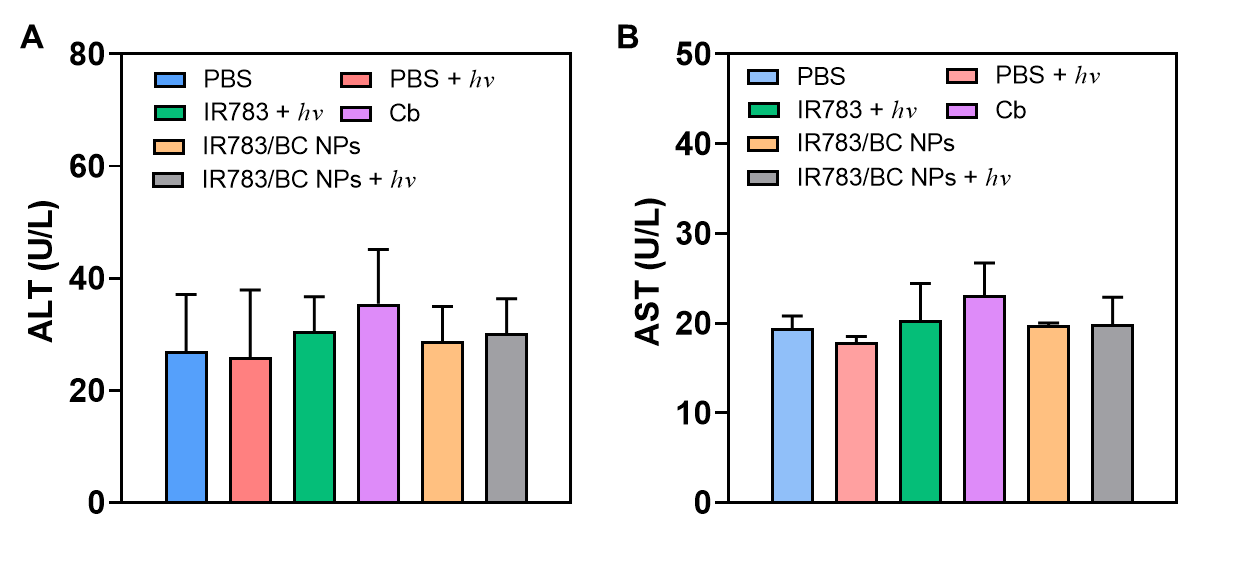


**Figure S21.** Evaluation of the secretion of alanine aminotransferase (ALT) and aspartate aminotransferase (AST) after different treatments.
